# A Computer Simulation Insight into the Formation of Apocarotenoids: Study of the Carotenoid Oxygenases BCO1 and BCO2 and Their Interaction with Putative Substrates

**DOI:** 10.3390/molecules27227813

**Published:** 2022-11-13

**Authors:** Anabela Martínez, Jorge Cantero, Antonio J. Meléndez-Martínez, Margot Paulino

**Affiliations:** 1 Bioinformatics Area, DETEMA Department, Faculty of Chemistry, UdelaR, Montevideo 11600, Uruguay; 2 Medical Research Center, Faculty of Health Sciences, Universidad Nacional del Este, Minga Guazu 7420, Paraguay; 3 Food Color and Quality Laboratory, Faculty of Pharmacy, Universidad de Sevilla, 41012 Sevilla, Spain

**Keywords:** carotenoid oxygenases, molecular dynamics, ligand interaction

## Abstract

Carotenoids are isoprenoid pigments, and sources of vitamin A in humans. The first metabolic pathway for their synthesis is mediated by the enzymes β,β-carotene-15,15′-dioxygenase (BCO1) and β,β-carotene-9′,10′-dioxygenase (BCO2), which cleave carotenoids into smaller compounds, called apocarotenoids. The objective of this study is to gain insight into the interaction of BCO1 and BCO2 with carotenoids, adding structural diversity and importance in the agro-food and/or health sectors. Homology modeling of BCO1 and BCO2, and the molecular dynamics of complexes with all carotenoids were performed. Interaction energy and structures were analyzed. For both enzymes, the general structure is conserved with a seven beta-sheet structure, and the β-carotene is positioned at an optimal distance from the catalytic center. Fe^2+^ forms in an octahedral coordination sphere with four perfectly conserved histidine residues. BCO1 finds stability in a structure in which the β-carotene is positioned ready for enzymatic catalysis at the 15–15′ bond, and BCO2 in positioning the bond to be cleaved (C9–C10) close to the active site. In BCO1 the carotenoids interact with only seven residues with aromatic rings, while the interaction of BCO2 is much more varied in terms of the type of interaction, with more residues of different chemical natures.

## 1. Introduction

Carotenoids are very versatile isoprenoid compounds of great interest in the agro-food, health and cosmetics sectors [1]. Evidence has accumulated in the last years that carotenoid derivatives usually termed as “apocarotenoids” could be involved in biological actions in humans [2]. However, the term is confusing as the IUPAC definition does not match the current usage of the term by scientists, who generally use it to designate carotenoid derivatives formed by cleavage [3]. Apocarotenoid compounds can be formed by oxidative cleavage of carotenoids through the action of carotenoid-cleavage oxygenases (CCO), a widely distributed family of enzymes with more than 200 possible variants, which differ from each other by substrate specificity and the position where they cleave the bond [4,5]. Retinal, from which other retinoids with vitamin A activity (retinoic acid, retinol) are formed, results from the cleavage of provitamin A carotenoids [6]. This is considered the vitamin with the broadest spectrum of actions in humans [3]. It is essential in processes of vision, reproduction, immunity, cell differentiation, apoptosis, maintenance of epithelial tissue, brain function and growth, embryonic development, etc. [3,6]. The main dietary provitamin A carotenoids are β-carotene, β-cryptoxanthin and α-carotene, which are usually obtained from plant foods and are ingested in amounts of a few mg/day [3,7]. The first step in the metabolism of vitamin A is the conversion of provitamin A carotenoids into retinal, a process that is carried out by β,β-carotene-15,15′-dioxygenase (BCO1). Another type of BCO is known in mammals, β,β-carotene-9′,10′-dioxygenase (BCO2). Both enzymes are mainly expressed in the intestine [8] and have the same mechanism of action: they cleave a carbon–carbon double bond and incorporate two oxygen atoms into the substrate [9,10]. BCO1, a cytoplasmic protein [8], cleaves β-carotene at the central 15–15′ double bond and is considered the main pathway in retinol synthesis [11]. On the other hand, BCO2 is expressed in the inner mitochondrial membrane [8] and can be considered an alternative pathway for retinal formation. BCO2 cleaves at positions 9–10 and 9′–10′ and has a broader substrate specificity [9,12,13]. The difference in cell compartmentalization is thought to be a possible key to the different substrate specificity between the two enzymes [11].

The substitution of a hydroxyl group by a hydrogen atom on the β-ring decreases the activity of BCO1 for the carotenoid, but not that of BCO2 [12]. For this reason, BCO1 shows less activity for β-cryptoxanthin than for β-carotene [14] and zero activity for zeaxanthin and lutein, while the same does not occur for BCO2 [9,14]. On the other hand, Carlo dela Seña et al. [14] reported for the first time that lycopene, even without a β-ring, shows high activity for BCO1.

There are over fifty carotenoids that possess at least one unsubstituted β-ring and can therefore exhibit provitamin A activity [12]. Apart from the three main dietary provitamin A carotenoids mentioned above (α-carotene, β-carotene and β-cryptoxanthin), the other major carotenoids found in human fluids and tissues are lutein, zeaxanthin, lycopene and the colorless carotenoids phytoene and phytofluene. The latter two have been traditionally overlooked but are among the major dietary carotenoids (as demonstrated in Luxembourg and Spain) and are eliciting increased interest in food science, nutrition and cosmetics [3,15]. The objective of this study is to gain insight into the interaction of the major carotenoids found in humans with BCO1 and BCO2 by in silico approaches. Altogether, those carotenoids differ considerably in structure (Table 1), making it possible to draw interesting conclusions about structure–interaction relationships. Additionally, other carotenoids adding structural diversity and importance in the agro-food and/or health sectors were considered. These were: violaxanthin, a carotenoid with two epoxide groups that is not present in plasma but is a major dietary carotenoid found in green vegetables and other plant foods [15,16,17]; astaxanthin and canthaxanthin, two ketocarotenoids bioavailable in humans, which are used as additives and can be found in feeds and foods, typically marine [1]. Two optical isomers of zeaxanthin were also studied, namely (3*R*-3′*R*)-zeaxanthin and (3*R*,3′*S*, *meso*)-zeaxanthin. Both are found in the macula lutea of the human retina, although (3*R*,3′*S*, *meso*)-zeaxanthin is not found in natural foods and is thought to be formed from lutein in the retina [18,19].

## 2. Results

### 2.1. Primary Structure

BCO1 is a five hundred and forty-seven amino acid residue protein with a molecular mass of 62.64 KDa. BCO2 is a five hundred seventy-nine amino acid residue protein with a molecular mass of 65.67 KDa. Their primary structure is shown in the supplementary material (Supplementary Materials Figures S1 and S2).

### 2.2. Search for Homologous Sequences

BLAST describes sixteen sequences that significantly align with BCO1 and the parameters: Max Score, Total Score, Query Cover, E Value and Percent Identity. A table with a summary of the main characteristics of the best-aligned sequences is attached; those with a percentage identity of less than twenty were discarded (Supplementary Materials Table S1). The SAS platform (Sequence Annotated by Structure) describes twenty-eight sequences that align with BCO1 and the parameters: percentage of identity, amino acid residues overlap, sequence length (Seq Ien) and E-value. A table with the parameters of the most relevant ones is attached (Supplementary Materials Table S2).

Most of the results are crystal structures of RPE65 expression in *Bos taurus*. RPE65 is only expressed in vertebrates and is considered a subgroup of CCOs. It catalyzes a concerted ester cleavage and trans-cis isomerization of all-trans-retinyl esters, and has also been shown to be the lutein to *meso*-zeaxanthin isomerase in the vertebrate eye [20]. RPE65 has an additional function as the lutein to *meso*-zeaxanthin isomerase in the vertebrate eye. Of all these, the crystal code PDB 4ryx was selected for homology modeling in MOE. It corresponds to a crystal structure of RPE65 in complex with the fatty acid palmitate and with Fe^2+^ in its catalytic center. This crystal has a resolution of 2.0 Å and a percentage identity with BCO1 of 37.8%, these being among the best values found. For BCO2, the same criteria were used as for BCO1, and similar results were obtained. In consequence, the 4ryx crystal was again selected.

### 2.3. Homology Modeling Results

Homology modeling performed in MOE with the 4ryx crystal as template gave eleven putative models and the parameters are reported (Supplementary Materials Tables S3 and S4): RMSD to mean (the heavy atom RMSD of each model to the average position of all of the intermediate models), RMSD measured between the α carbons (CA RMSD to mean), Contact Energy (desolvation free energy needed to transfer atoms from water to the interior of the protein), Packing Score, GB/VI (generalized Coulomb and Born interaction energy), Total Potential Energy (U), Solvation Energy (E sol), Electrostatic Energy (E elec), Van der Waals Energy (E vdw) and Total bonded Energy (E bond).

The program modeled, in the case of BCO1, five hundred twenty amino acid residues out of five hundred and forty-seven in the primary structure, and in the case of BCO2, five hundred thirty-six amino acid residues out of five hundred and seventy-nine in the primary structure. All amino acid residues that remained unmodeled correspond to N- or C-terminal regions. After an analysis of the initial structure (obtained from the gene Q9HAY6 from BCO1 and Q9BYV7 from BCO2) and final (3D structure) sequences (Supplementary Materials Figures S3 and S4) we concluded that the missing regions are not relevant for the study proposed here.

For BCO1, the selected model (the one with the lowest potential energy), has an RMSD of 0.5 Å and an RMSD of CA is 0.31 Å.

For BCO2, it has an RMSD of 0.66 Å and an RMSD measured between the α carbons of 0.56 Å.

The superposition of the secondary structures of the models obtained by homology modeling of BCO1 and BCO2, and the percentages of secondary structures for both proteins are shown in the supplementary material (Supplementary Materials Figure S5 and Table S5).

### 2.4. Molecular Dynamics of β-carotene-BCOs

#### 2.4.1. General Structure and Active Sites

**Figure 1:**
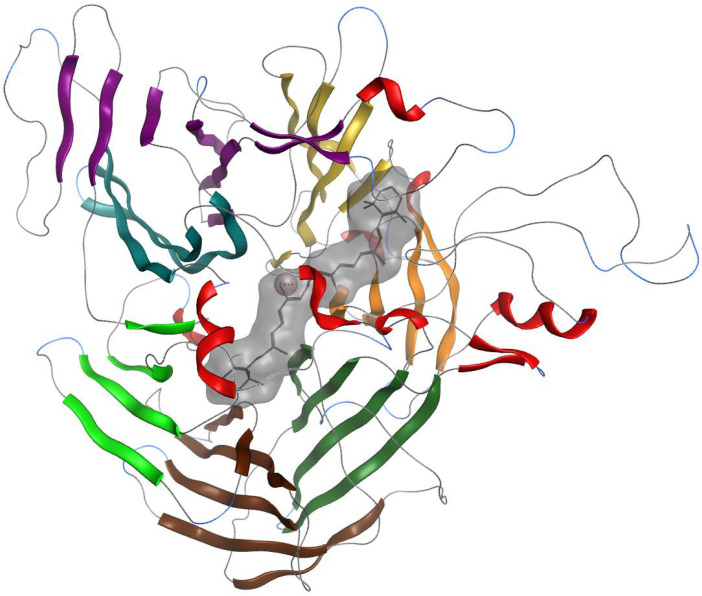
Three-dimensional ribbon display of the BCO1 structure. The beta-sheets colored in light green, light blue, violet, mustard, orange, dark green and brown can be distinguished. The alpha helices are shown in red, and the loops are shown in thin gray lines. The representation of the ligand in gray rods and of the metal in pink is shown in the center.

For both enzymes, we positioned the β-carotene manually: We superimposed our model with the 2biw crystal structure, which has an apocarotenoid as a ligand and performs a symmetric cleavage (15–15′). With this, we defined the binding site and the appropriate pose, and manually reconstructed β -carotene from this structure, respecting the crystallographic position of the substrate. This model with β -carotene as a ligand was subjected to 100 ns molecular dynamics. The modeling time (time of the simulation) for evaluation of the catalytic center distance was assessed as 100 ns for two reasons. Firstly, 100 ns is a generally accepted standard time for conformational sampling. Secondly, the thermodynamic equilibrium was reached after 5 ns of simulation, as may be verified in Supplementary Materials Figures S8–S15, validating the 100 ns selection. After 100 ns of simulation, a seven beta-sheet structure is observed. The β-carotene is shown in the pocket at an optimal distance from the catalytic center. Figure 1 shows this structure for BCO1.

**Figure 2:**
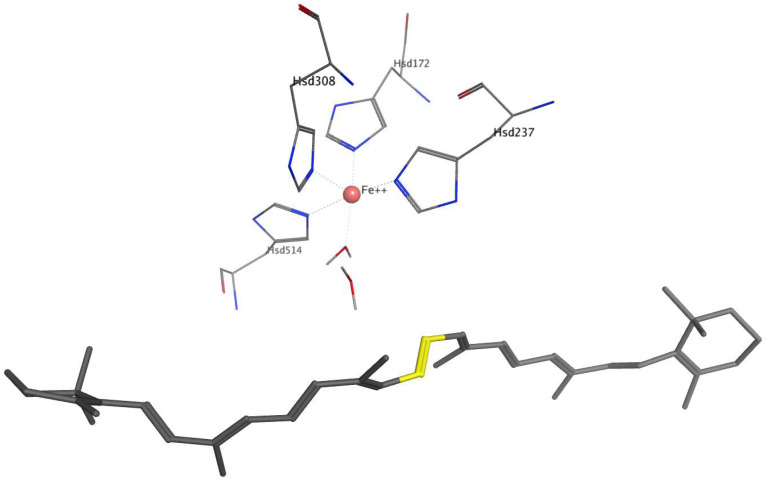
Two-dimensional representation of the β-carotene (gray rods, and in yellow the 15–15′ bond) and the coordination center, for BCO1. The Fe^2+^ atom, represented by a pink sphere, coordinates with His172, His237, His308, His514 and two water molecules (TIP2089 and TIP2035), all represented by thin rods and colored according to atom type. The average bond distances (in Å) between Fe^2+^ and the histidine nitrogens are 2.030 ± 0.075, 2.08 ± 0.084, 2.11 ± 0.096 and 2.12 ± 0.11, respectively. Between Fe^2+^ and the oxygens of water, the distances are 1.97 ± 0.079 and 1.94 ± 0.067, respectively.

**Figure 3:**
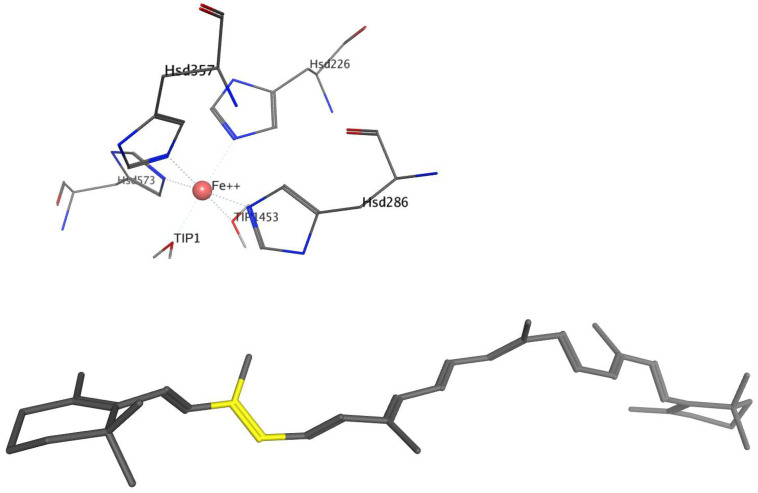
Rod representation of the active site of BCO2 and β-carotene. The Fe^2+^ atom, represented by a pink sphere, coordinates with Hys226, Hys286, Hys357, Hys573 and two water molecules (TIP1, TIP1453), all represented by thin rods and colored according to atom type. The β-carotene is represented by gray rods, and the 9–10 bond is in yellow. The average bond distances in Å between Fe^2+^ and the histidine nitrogens are 2.09 ± 0.095, 2.09 ± 0.090, 2.07 ± 0.080 and 2.11 ± 0.094, respectively, and between Fe^2+^ and the oxygens of the waters, 1.96 ± 0.071 and 1.95 ± 0.064, respectively.

Figure 2 and Figure 3 show β-carotene and Fe^2+^ with an octahedral coordination sphere with the four perfectly conserved histidine residues, and two water molecules, for BCO1 and BCO2 respectively.

For BOCO1, Table 2 presents the average bond distances of the 15, 15′, 14 and 14′ carbons of β-carotene to Fe^2+^, and the distances of these β-carotene atoms to the two water molecules forming part of the Fe^2+^ coordination center (in a putative place that could be occupied by oxygen in the catalytic mechanism).

For BCO2, the coordination sphere is not centered on the 15–15′ bonds but is closer to the β-ring. Table 3 presents the average bond distances between the Fe^2+^ and of the two water molecules to the β-carotene atoms closest to these, i.e., C8, C9, C10 and C11.

For more information, plots of the fluctuations of these distances throughout the entire production step are shown in the supplementary material (Supplementary Materials Figures S6 and S7).

As shown in Table 2, the distance from the ligand to the catalytic center of BCO1, measured between TIP2035 water and the 15 and 15′ atoms of β-carotene, is approximately 4.5 Å. The enzymatic cutoff that β-carotene undergoes is at 15–15′, so we assume that the water molecule would be located at the molecular oxygen site in the catalytic mechanism.

On the other hand, atoms 15 and 15′ are the closest to Fe^2+^ at a distance of approximately 6.5 Å.

For BCO2 it is reported that β-carotene is cleaved at the C9–C10 bond. In our study, we confirm that report albeit with a subtle difference: we found that the optimal distance from the ligand to the active site between the water molecule TIP1 and atoms C8 and C9 of β-carotene, is approximately 6 Å. On the other hand, the distance between these atoms and Fe^2+^ is approximately 7.5 Å. Anyway, the distance from TIP1 and Fe^2+^ to C10 is 6.75 and 8.17, respectively, being only 0.9 and 0.6 Å, respectively, larger than for C8.

#### 2.4.2. Energy and Structure Analysis

The plots of the total energy, RMSD and RMSF of the system evaluated for protein, ion, ligand and solvent, and of the interaction energy of the protein and solvent with the ligand, along the 100 ns molecular dynamics trajectory, are attached in the supplementary material (Supplementary Materials Figures S8–S10 and S12–S14). Also attached are plots of the different components contributing to the total energy, both kinetic and potential, calculated in the force field terms (bond, angles, dihedral, improper, electrostatic and Van der Waals) (Supplementary Materials Figures S11 and S15).

For BCO1, the mean total potential energy of the system is −180,376.10 ± 307.88 Kcal.mol^−1^, and in BCO2 it is −172,277.19 ± 292.90 Kcal.mol^−1^. We do not consider that this energetic difference can provide information about the stability of the enzymes, since these are systems that differ in the number of atoms.

As for the RMSD, for the molecular dynamics trajectory in BCO1, the average is 2.89 ± 0.36 Å and for BCO2 it is 3.09 ± 0.33 Å.

That is, the average values for both structures are approximately the same, being only 0.2 Å larger for BCO2.

From the figures in the supplementary material, we observe that for BCO1 stability is reached after 20 ns of simulation, although it continues to grow slightly throughout the simulation and has some parts that rearrange, which is characteristic of the structure. In the case of BCO2, stability is reached at about 10 ns and remains constant with parts that rearrange but do not grow.

With respect to RMSF, it is observed that for both enzymes, residues with RMSF > 2 Å appear in areas of undefined secondary structure, in loops or in areas where the protein is highly exposed to the solvent.

#### 2.4.3. Interaction with β-carotene

The average potential interaction energy of BCO1 with β-carotene (Uab) is −94.78 ± 3.84 Kcal.mol^−1^, −6.04 ± 2.19 Kcal.mol^−1^ and −88.76 ± 3.09 Kcal.mol^−1^, for the total potential interaction energy, the electrostatic and Van der Waals components of the interaction energy, respectively. The average potential interaction energy of BCO2 with β-carotene (Uab) is −91.74 ± 3.99 Kcal.mol^−1^, −5.82 ± 2.22 Kcal.mol^−1^ and −85.92 ± 3.49 Kcal.mol^−1^, for total energy, electrostatic and VDW component, respectively. The plots of Uab are attached in the supplementary material (Supplementary Materials Figures S16 and S18)

**Figure 4:**
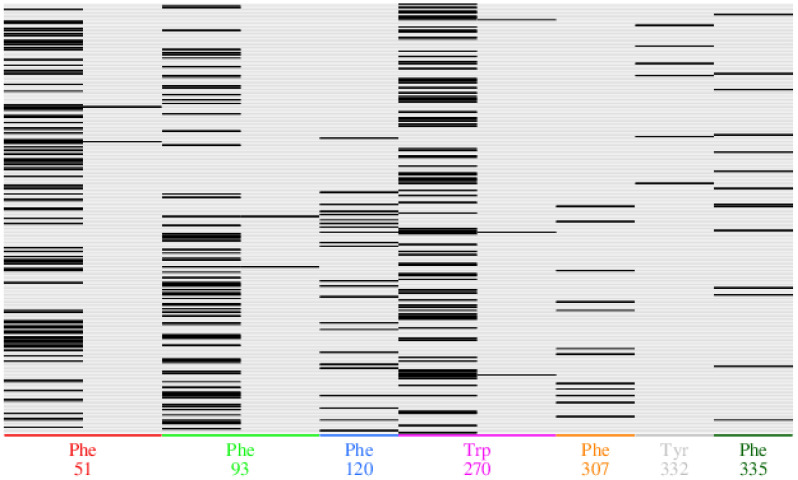
Results of fingerprinting (PLIF) analysis for the interaction of BCO1 with β-carotene. Fingerprints are shown for each residue of the ligand-interacting protein. Each line represents an interaction of the due residue with one atom of the ligand at 100 ns of simulation.

**Figure 5:**
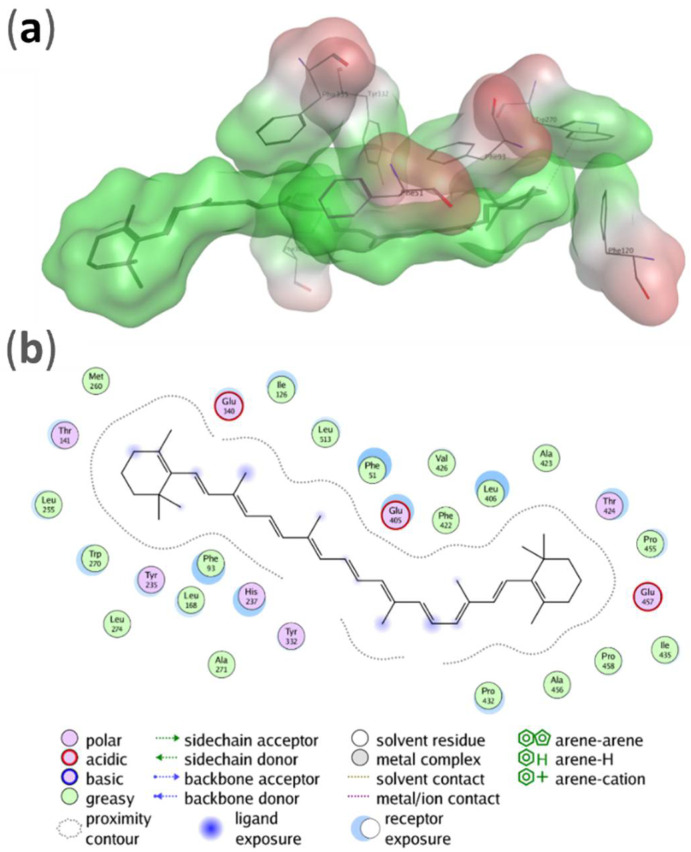
(**a**) Potential energy surface over the β-carotene and the BCO1 residues with which it interacts, according to PLIF analysis. The hydrophobic areas are colored in green and the hydrophilic areas in red. (**b**) Two-dimensional representation of the interactions established by β-carotene within the BCO1 catalytic site. Only the residues contacting the carotenoid at less than 4.5 Å distance are shown.

**Figure 6:**
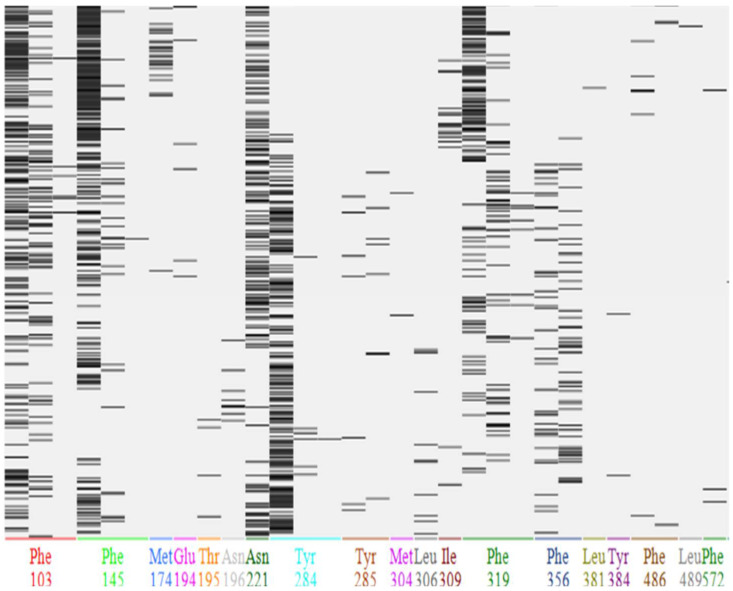
Results of PLIF analysis for the interaction of BCO2 with β-carotene. Fingerprints are shown for each residue of the ligand-interaction protein. Each line represents an interaction of the due residue with one atom of the ligand at 100 ns of simulation.

**Figure 7:**
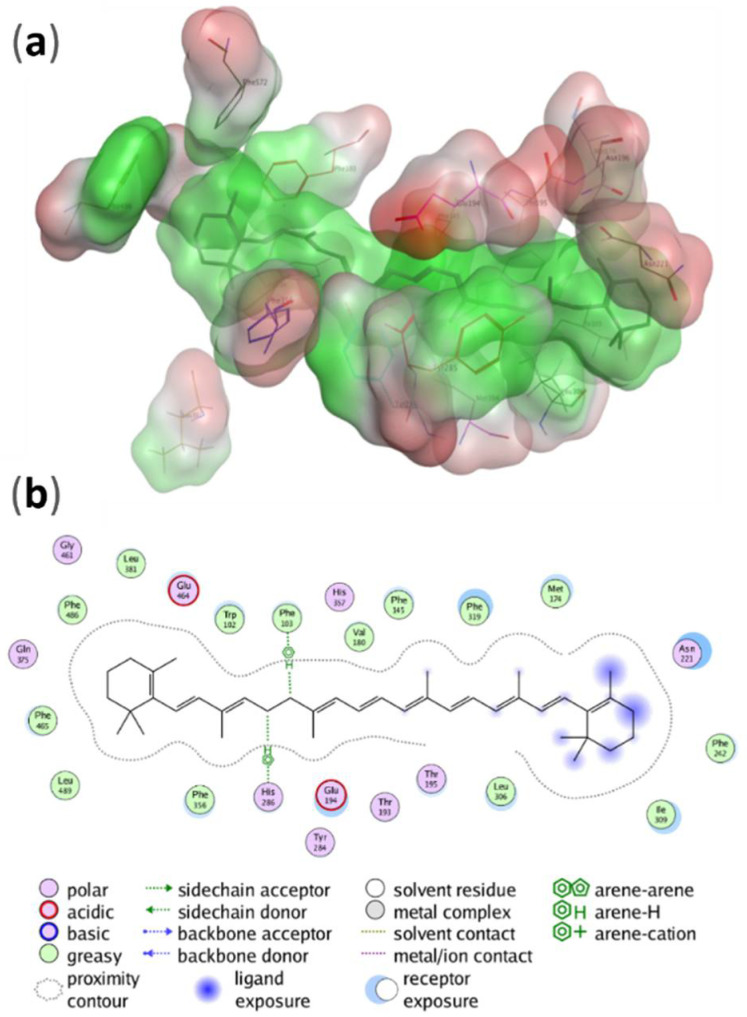
(**a**) Potential energy surface over the β-carotene and the BCO2 residues with which it interacts, according to PLIF analysis. With green the hydrophobic areas and with red the hydrophilic areas. (**b**) Two-dimensional representation of the interactions established by β-carotene within the BCO2 catalytic site. Only the residues contacting the carotenoid at less than 4.5 Å distance are shown.

A fingerprinting scheme, (protein–ligand interaction, PLIF) for ligand interactions with different protein residues is shown in Figure 4 and a 3D scheme of these interactions, for BCO1, is shown in Figure 5. Figure 6 and Figure 7 show the PLIF scheme and the 3D scheme, respectively, for BCO2. The statistical analysis of the interaction frequency is attached in the supplementary material (Supplementary Materials Figures S17 and S19).

As can be deduced from Figure 4, β-carotene interacts through two atoms with Phe51, Phe93 and Trp270, and through one atom with Phe120, Phe307, Tyr332 and Phe335. Phe51 interacts 35.8% of the time, this residue having the highest interaction frequency. Phe93 and Trp270 also have a high interaction frequency. 

As shown in Figure 6, for BCO2, β-carotene interacts through three atoms with Phe103, Phe145, Phe319 and Tyr284, through two atoms with Phe486 and Tyr284, and through one atom with Met174, Glu194, Thr195, Asn196, Asn221, Met304, Leu306, Ile309, Leu381, Tyr384, Leu489 and Phe572.

On the other hand, Phe145 is the residue with the highest interaction frequency, 45.8%. Phe103, Asn221, Tyr284 and Phe319 also have a high interaction frequency.

From the PLIF analysis, we observe that BCO1 interacts with only seven residues during the 100 ns of simulation. Five of these are non-polar aromatic phenylalanines. The other two residues are tryptophan and tyrosine, also aromatic amino acid residues but polar in this case. Two of the phenylalanines and the tryptophan interact with the ligand through two atoms and have a high frequency of interaction with one of these atoms, the highest frequency being 35.6% for Phe51.

### 2.5. Interaction of BCO1 with Other Carotenoids

After 100 ns of simulation of the BCO1 complex with β-carotene, trajectories of 1 ns of molecular dynamics per ligand were performed. For all models subjected to 1 ns molecular dynamics, we observed that the overall structure of the enzyme–ligand complex remains the same as in the complex with β-carotene. The coordination sphere with four histidine residues and two water molecules of the catalytic center is conserved. The 15 and 15′ atoms of the carotenoids remain the closest to Fe^2+^ and TIP2035 water.

Table 4 describes the potential interaction energy of the different carotenoids that underwent analysis with the rest of the system (protein and solvent).

Table 5 shows the fingerprint analysis (PLIF) for the interaction of all possible ligands with the protein residues and the water molecules with which they interact at the different times of production, for 1 ns of simulation.

All carotenoids interact with Phe51, with a high interaction percentage for the linear carotenoids (15*Z*)-phytofluene and (15*Z*)-phytoene. Phe51 is the residue with the highest interaction percentage for the reference molecule β-carotene. For β-carotene, other residues with a high interaction percentage are Trp270 and Phe93 (Figure 4). Both residues interact with all ligands with the exception of (15*Z*)-phytofluene and (15*Z*)-phytoene; furthermore, Phe93 is the residue with the highest interaction percentage for three ligands and interacts with two atoms of the ligand for eight of these carotenoids. The remaining phenylalanines that interact with β-carotene (120, 307 and 335) also interact with between ten and eleven other carotenoids.

All of these residues are aromatic, and accompany the interaction of a hydrophobic ligand with conjugated double bonds. This fact also agrees with the most important contribution being that of Van der Waals energies.

α-Carotene presents an interaction similar to that of β-carotene and the potential interaction energy between both of them differs by approximately 2 Kcal.mol^−1^ (Table 4 and Table 5).

(3*R*)-β-Cryptoxanthin and (3′*R*)-β-cryptoxanthin interact with the same phenylalanine residues, with at least one non-aromatic, non-polar residue, and with Trp270. They differ in that (3′*R*)-β-cryptoxanthin interacts with a positively charged residue (Arg408) and does not interact with the solvent, whereas (3*R*)-β-cryptoxanthin interacts with a neutral polar residue (Cys273) and with twenty-one water molecules, suggesting that the position of the carotenoid in the pocket mainly changes the interaction with the solvent.

The interactions of (all-E)-lycopene, (5*Z*)-lycopene and (5′*Z*)-lycopene are very similar; furthermore, the complex–ligand interaction energies (represented in Table 4 and Table 5) among the three do not differ by more than 3 Kcal.mol^−1^. This might suggest that the enzyme will not have specificity depending on whether the linear carotenoid is (all-E) or has a *Z* configuration at the C5-bond, or on which side the isomerized carotenoid is positioned.

Unlike the other major dietary carotenoids bioavailable in humans (lutein, zeaxanthin, β-cryptoxanthin, α-carotene, β-carotene and lycopene) the cleavage of phytoene and phytofluene by CCOs has not been described and cannot be presumed due to their markedly different degree of unsaturation, hence the interest in including them in this study. Phytoene and phytofluene differ in the presence of a double bond and have similar hydrophobic interaction, but differ in the interaction with polar and charged residues. Phytofluene, which has a double bond at bond eleven, interacts with Tyr332, Tyr235, Tyr326 and His237, while phytoene, which has a single bond at bond eleven, interacts with His237 and Arg408. Phytofluene, besides having more polar interactions, has a potential interaction energy of approximately 10 Kcal.mol^−1^ less, this difference being more accentuated in the Van der Waals contribution.

(3*R*,3′*S*, *meso*)-Zeaxanthin and (3*R*,3′*R*)-lutein, two positional isomers, which differ in the position of the double bond of one of the β-rings, have very similar interaction with residues and potential interaction energy. Both interact with the same number of water molecules and with the same residues, only (3*R*,3′*R*)-lutein additionally interacts with Phe307, Trp270 and His237 and Pro455 while (3*R*,3′*S*, *meso*)-zeaxanthin interacts with Ala456. (3*R*,3′*R*)-Zeaxanthin, an optical isomer of (3*R*,3′*S*, *meso*)-zeaxanthin that differs in the orientation of one of the OH^-^, has a potential interaction energy with the protein of about 10 Kcal.mol^−1^ less, the main difference coming from the electrostatic contribution. (3*R*,3′*R*)-Zeaxanthin interacts with twelve fewer water molecules, but interacts with three neutral polar residues (Thr141, Thr424 and Tyr332), two negatively charged residues (Gln457 and Asp12) and one positively charged residue (Arg408), with which (3*R*,3′*S*, *meso*)-zeaxanthin does not interact. Furthermore, the residue with the highest interaction frequency for (3*R*,3′*R*)-zeaxanthin is the polar residue Thr141, with a frequency of 96%, while for (3*R*,3′*S*, *meso*)-zeaxanthin it is the non-polar residue Ala456, with a frequency of 85%. All this suggests to us that the position of the double bond in one of the β-rings will not significantly change the preference of the enzyme for one ligand or the other, whereas the orientation of one of the hydroxy groups may generate more significant changes with respect to the interaction.

(3*S*,3′*S*)-Astaxanthin is the ligand with the best potential interaction energy (Uab), with −124 Kcal.mol^−1^, followed by (3*R*,3′*R*)-zeaxanthin with −116 Kcal.mol^−1^. This energy difference with the rest of the carotenoids comes mainly from the electrostatic contribution. This is justified since astaxanthin has an alcohol group and a ketone group on the two β-rings. This ligand presents interaction with four non-polar residues, with three aromatic non-polar residues, with two polar residues, with one aromatic polar residue and with thirty water molecules.

Canthaxanthin has a ketone group on the two β-rings like astaxanthin, but differs from astaxanthin in that it does not have alcohol groups. This carotenoid interacts with the same aromatic residues (polar and non-polar) as (3*S*,3′*S*)-astaxanthin, but only interacts with one polar and one non-polar non-aromatic residue, and with twenty-three water molecules. It also interacts with one histidine residue. Canthaxanthin has a potential interaction energy approximately 16 Kcal.mol^−1^ higher than (3*S*,3′*S*)-astaxanthin, the difference coming from the electrostatic contribution.

(3*S*,3′*S*)-Violaxanthin has an alcohol group and an epoxy group present on both β-rings. This ligand interacts with fifteen residues, being the carotenoid with the highest number of interactions along with zeaxanthin. The residues with which it interacts are of all chemical natures: non-polar, aromatic non-polar, polar, aromatic polar, negatively charged and positively charged. It interacts with a single water molecule. It is the only ligand that presents a higher frequency of interaction with a charged residue, being 58% with arginine. It is also the only carotenoid that interacts with a residue through four atoms of the ligand (with threonine). With respect to the system–ligand binding energy, it is the carotenoid with the highest energy of those presenting two alcohol groups, having a very similar contribution to that of β-cryptoxanthin. Considering its structure, such changes may be attributed to the presence of the epoxide groups.

In summary, three groups of carotenoids could be arbitrarily established in terms of Uab values:

- Carotenes (α- and β-carotene, lycopene, phytoene and phytofluene) with Uab values in the interval ~−93 and −102 Kcal.mol^−^^1^. It should be noted that they differed clearly in structure (from none to two end rings, from three to eleven conjugated double bonds and with diverse geometrical configurations), which suggests that such differences in carotenes do not lead to energetic differences over teen units.
- Carotenoids with one hydroxy group (β-cryptoxanthin), two hydroxy groups (lutein, zeaxanthin), two hydroxy groups and two 5,6-epoxide groups ((3*S*,3′*S*)-violaxanthin) and two ketone groups ((3*S*,3′*S*)-astaxanthin) (all such groups in rings), with Uab values in the range ~−100 to −116 Kcal.mol^−^^1^.
- Carotenoids with one hydroxy and one ketone group in each terminal ring ((3*S*,3′*S*)-astaxanthin), with the lowest Uab value (~−124 Kcal.mol^−^^1^).

### 2.6. Interaction of BCO2 with Other Carotenoids

As in BCO1, we observe that the overall structure of the enzyme–ligand complex, for all models subjected to 1 ns molecular dynamics, remains the same as in the complex with β-carotene.

Table 6 and Table 7 show the interaction energies and fingerprint analysis, respectively, for the same ligands as in BCO1.

Again, three groups could be arbitrarily established considering Uab values:

- Carotenes (α- and β-carotene, lycopene, phytoene and phytofluene) with Uab values in the interval ~−86 to −104 Kcal.mol^−^^1^.
- Carotenoids with one hydroxy group (β-cryptoxanthin), two hydroxy groups (lutein, zeaxanthin), two hydroxy groups and two 5,6-epoxide groups ((3*S*,3′*S*)-violaxanthin) and two ketone groups ((3*S*,3′*S*)-astaxanthin) (all such groups in rings), with Uab values in the range ~−104 to −118 Kcal.mol^−^^1^.
- Carotenoids with one hydroxy and one ketone group in each terminal ring ((3*S*,3′*S*)-astaxanthin), with the lowest Uab value (~−127 Kcal.mol^−^^1^).

In general, the differences in Uab values between BCO1 and BCO2 were below 5 Kcal.mol^−1^, except for α-carotene, (3*R*′)-β-cryptoxanthin, (3*R*,3′*S*, *meso*)-zeaxanthin, (3*R*,3′*R*)-lutein, (3*S*,3′*S*)-violaxanthin and (15*Z*)-phytofluene, the latter two with differences > 10 Kcal.mol^−1^.

For BCO2, all carotenoids interact with Phe103. Fourteen of the fifteen carotenoids interact with Phe319, Phe145 and with Phe486. Thirteen of fifteen carotenoids interact with Phe356. In addition, Phe319 is the residue with the highest interaction frequency for five of these carotenoids.

Phe145, Phe103 and Phe319 are some of the residues that interact most frequently with the reference molecule β-carotene. Unlike BCO1, in BCO2 the residues interacting with β-carotene differ significantly from those interacting with the rest of the ligands.

α-Carotene is the carotenoid with the highest potential interaction energy, which is −86.56 ± 3.89 Kcal.mol^−1^ with a very small electrostatic contribution. All the residues with which it interacts are aromatic. It has a similar interaction than linear carotenoids (lycopene, phytoene and phytofluene), differing from these in the contribution of the polar residue tryptophan.

With respect to (all-E)-lycopene and its isomers, we note that only (5′*Z*)-lycopene has an interaction similar to that of (all-E)-lycopene, while (5*Z*)-lycopene interacts with significantly fewer residues. On the other hand, (5*Z*)-lycopene is the one with a potential interaction energy very similar to (all-E)-lycopene, while (5′*Z*)-lycopene has a potential interaction energy approximately 8 Kcal.mol^−1^ higher. This differs from BCO1 in which the position of isomerization did not change the interaction.

With respect to (15*Z*)-phytoene and (15*Z*)-phytofluene, these have a similar interaction, the main difference being that phytoene interacts with two negatively charged residues (His286 and His357), whereas phytofluene does not interact with any charged residues. The potential interaction energy for both is also similar, being approximately 3 Kcal.mol^−1^ higher for (15*Z*)-phytofluene, with this difference coming from the Van der Waals contribution. This is different from that observed for BCO1 where both molecules differ in their interaction more significantly.

(3*R*)-β-Cryptoxanthin and (3*R*′)-β-cryptoxanthin have a very similar non-polar interaction, and both have a higher frequency of interaction with the same aromatic non-polar residue (Phe145). As in BCO1, they differ in polar interaction. While (3*R*)-β-cryptoxanthin interacts with one neutral polar residue, one positively charged residue and forty-one water molecules, (3*R*′)-β-cryptoxanthin interacts with three negatively charged residues and sixteen water molecules. With respect to energy, (3*R*′)-β-cryptoxanthin has an electrostatic contribution approximately 4 Kcal.mol^−1^ higher than (3*R*)-β-cryptoxanthin. As for BCO1, we suggest that the position of the double bond mainly changes the hydrophilic interaction.

With respect to the isomers (3*R*,3′*R*)-zeaxanthin, (3*R*,3′*S*, *meso*)-zeaxanthin and lutein, in BCO2, no significant differences were observed with respect to their interactions. The interaction energies do not differ among them by more than 2 Kcal.mol^−1^ for each component. They also show similar interactions with residues and solvent.

As in BCO1, (3*S*,3′*S*)-astaxanthin is the ligand with the best potential interaction energy, being −126.90 Kcal.mol^−1^, with an electrostatic contribution of −38.21 Kcal.mol^−1^. This carotenoid interacts with seven non-polar residues, with only one polar residue and one negatively charged residue, and with twenty-four water molecules, and presents the highest interaction frequency with one of them.

Canthaxanthin interacts with six non-polar residues, with one polar residue, with one positively charged residue, with three negatively charged residues and with forty-seven water molecules. It has a significantly higher potential interaction energy than (3*S*,3′*S*)-astaxanthin, with an approximately 16 Kcal.mol^−1^ higher electrostatic contribution and a 6 Kcal.mol^−1^ higher Van der Waals contribution.

As in BCO1, (3*S*,3′*S*)-violaxanthin is the carotenoid that interacts with the greatest number of residues (fourteen), these being of all chemical natures. It is also the carotenoid that interacts with the largest number of water molecules (sixty-two). It has a potential interaction energy of −118.04 Kcal.mol^−1^, with an electrostatic contribution of −33.96 Kca.mol^−1^. It differs from BCO1, where it is the carotenoid with the highest potential interaction energy of those with two alcohol groups.

As seen in Table 4 and Table 6, the differences in conformational energies at the minimum and at the current pose are always lower in absolute value than the interaction energy Uab. This ensures that the interaction energy is sufficient to compensate for the energetic cost required by the pose adopted by the ligand at the site.

## 3. Materials and Methods

### 3.1. Modeling of BCO1 and BCO2

To carry out this work, we started with the primary structure of proteins. Data were extracted from the UniProt protein database [21].

Three-dimensional modeling was performed with the homology modeling technique, using the MOE program version 2015 [14], and the Amber 12 force field [22]. The template for the homology modeling was searched on the Sequence Annotated by Structure (SAS) [23] and Basic Local Alignment Search Tool (BLAST) [24] software. BLAST is a local sequence alignment software and is able to compare a query sequence against a large number of sequences in a database. The algorithm finds the sequences in the database that are most closely related to the query sequence to infer evolutionary relationships between sequences and identify gene family members. The crystal structures of the possible templates were searched in the Protein Data Bank (PDB) database [25].

To select the best template, we analyzed their evolutionary character, their functionality, on which organism and type of tissues they are expressed, the type of reaction they catalyze, and which ligands they use. Seeking to find the greatest similarity in these data between our protein to be modeled and our template.

After homology modeling with the selected template, we obtained eleven possible structures, from which we selected the one with the lowest total potential energy (U) in Kcal.mol^−1^.

For the selected model, we proceeded to position the ligands. For both enzymes, a plan was developed in which the first stage consisted of studying the interaction with β-carotene by anchoring and molecular dynamics, and then using the model obtained as a basis for extending the investigation to other carotenoids of interest.

### 3.2. Anchorage of β-carotene to BCO1 and BCO2

For the first stage, β-carotene was positioned within the active site of the enzymes. The β-carotene was chosen as the reference molecule because it is the molecule with the highest pro-vitamin A activity.

The β-carotene was positioned manually, using all the information on such positioning. In the MOE program, the structure of the BCO models was superimposed with a crystallized structure with a fatty acid as a ligand and its pose was copied into our model. It was then modified to become β-carotene. The choice of the reference crystal was made based on the literature data, considering it to be within the enzyme family, and the position of the bond they cleave.

To obtain a good starting structure for the molecular dynamics, after several trials, a five-step minimization protocol was established to be performed in MOE: First, the ligand alone was minimized. Second, a minimization was performed in a 4.5 Å coordination sphere around the ligand, leaving the rest of the system fixed, that is to say, the ligand, the Fe^2+^ and the four-histidine coordination sphere. Third, the same sphere was taken but included the ligand and Fe^2+^ along with its coordination sphere. Fourth, the whole system was minimized with restriction on the backbone, and finally, the whole system was without any restriction. All minimizations were performed with the AMBER 12: EHT force field [22] until a gradient of 0.00001 Kcal.mol^−1^Å^−2^ was reached.

### 3.3. Molecular Dynamics of BCOs–β-carotene Complexes

These models were subjected to explicit solvent molecular dynamics with a 15 Å water box. NaCl salt concentration of 0.15 M was used, using the NAMD program v2.13 with CUDA [26] and the CHARMM36 force field [27]. Table 8 details the protocol used.

The trajectories obtained were analyzed in order to study the thermodynamic, structural and geometrical stability of the protein, as well as the interaction with the ligand.

The main interest was in bond distances, total potential energy terms (U), root mean square deviation (RMSD), root mean square fluctuation (RMSF), ligand potential interaction energy (Uab) and protein–ligand interaction fingerprints (PLIF).

### 3.4. Complex Construction with the Ligands and 1 ns Dynamics

The models obtained after 100 ns of simulation with β-carotene were used as starting models to construct enzyme–ligand complexes with the carotenoids: α-carotene, β-cryptoxanthin, (3*R*,3′*R*)-zeaxanthin, (3*R*,3′*S*, *meso*)-zeaxanthin, lutein, (3*S*,3′*S*)-astaxanthin, (3*S*,3′*S*)-violaxanthin, canthaxanthin, (all-E)-lycopene, (5*Z*)-lycopene, (5′*Z*)-lycopene, (15*Z*)-phytofluene and (15*Z*)-phytoene (Table 1).

All these complexes were subjected to 1 ns molecular dynamics in NAMD with the CHARMM36 force field. Molecular dynamics consists of 1 ns of production, in 500 sampling steps. It has previous stages of minimization, heating and equilibration with the same protocol described in Table 8.

The ligand potential interaction energy (Uab) was calculated on these trajectories and the protein residues interacting with the ligand were analyzed using the protein–ligand fingerprint (PLIF) method. The PLIF descriptors implemented in the MOE were used as a benchmark for the interaction fingerprints. The interactions are classified as hydrogen bonds, ionic interactions and surface contacts according to the residues which are representative of a given set of structures of the protein–ligand complexes. The PLIF descriptors for all protein–ligands for the MD trajectory were generated with the default parameter set in MOE and presented in the table of fingerprints (Table 5 and Table 7) and the barcode display. The analysis was performed taking the collection of structures emerging from the molecular dynamics simulations through the 100 analyzed nanoseconds. This means that the interactions were analyzed by means of the statistical tools developed for the creation of each fingerprint.

## 4. Conclusions

### 4.1. General Structure

For both enzymes the general structure of carotenoid cleavage enzymes is conserved with a seven beta-sheet structure, and the β-carotene positioned at an optimal distance from the catalytic center. Fe^2+^ forms an octahedral coordination sphere with four perfectly conserved histidine residues, and two water molecules.

The distance between a water molecule found in the catalytic center of BCO1 and the 15 and 15′ carbon atoms of β-carotene was approximately 4.5 Å. As the enzymatic cutoff that β-carotene undergoes is at 15–15′, we assumed, on one hand, that the water molecule would be located at the molecular oxygen site participating in the biocatalytic mechanism. On the other hand, the carbon atoms 15 and 15′ were the closest to Fe^2+^, at a distance of approximately 6.5 Å.

The results obtained for BCO2 confirmed the reported data indicating a cleavage of β-carotene at the C9–C10 bond. An optimal distance from the ligand to the active site between the water molecule TIP1 and atoms C8 and C9 of β-carotene was approximately 6 Å, and 7.5 Å from Fe^2+^. For BCO2, we can conclude that it finds a good energetic stability with respect to β-carotene by positioning the bond to be cleaved (C9–C10) close to the active site, although not exactly on it, but slightly shifted toward the β-ring. Moreover, the bond distances are longer than in BCO1. This may be related to the reported literature data [9,12,13] that BCO2 is an alternative pathway for catalysis, with BCO1 being the predominant enzyme for cleavage.

### 4.2. Interaction of BCO1 and BCO2 with β-carotene

It can be seen that, while in BCO1 the β-carotene interacts with only seven residues, all of them with aromatic rings, the interaction of BCO2 is much more varied in terms of the type of interaction. BCO2 interacts with nineteen residues of different chemical natures and presents more hydrophobic interactions. Unlike BCO1, BCO2 interacts with nineteen residues: six phenylalanines, three tyrosines, three leucines, two methionines, two asparagines, a glutamine, a threonine and an isoleucine. That is, it interacts with non-polar, aromatic non-polar, polar, aromatic polar and negatively charged residues. With three phenylalanines and a tyrosine, it interacts through three atoms and through two atoms with another phenylalanine and another tyrosine. Of these four phenylalanines, the tyrosine that interacts through three atoms and one of the asparagine present a high frequency of interaction, being the highest at 45.8% for phenylalanine.

### 4.3. Interaction of BCO1 and BCO2 with other carotenoids

The results indicate that the xanthophylls (oxygenated carotenoids) had lower Uab values relative to carotenes (hydrocarbons) and that the presence of two hydroxy and two ketone groups in the ring ((3*S*,3′*S*)-astaxanthin) was associated to the lowest Uab values. It is noteworthy that the type and not just the number of oxygenated functions is important to explaining the Uab values, as (3*S*,3′*S*)-violaxanthin (also with four oxygenated functions in total, like (3*S*,3′*S*)-astaxanthin) had Uab values closer to the carotenoids with one or two oxygenated functions.

As a global observation, in the cases where there are alcohol groups, there is an increase in the electrostatic contribution. While the literature data show that a substitution of a hydroxyl group on the β-ring decreases the activity of BCO1, we looked at the binding energy, i.e., at the first step in the putative catalytic event. In order to reach a conclusion that proves the report we would have to consider the complete catalytic process and amplify this study using other methodologies, such as the recognized reaction path studies that can be performed by quantum mechanics, or other alternatives, such as hybrid methods that combine force fields with quantum mechanics.

## Figures and Tables

**Table 1:** Structural formulae of carotenoids that were analyzed.

β-Carotene	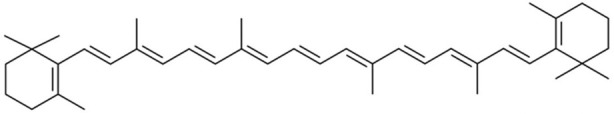
α-Carotene	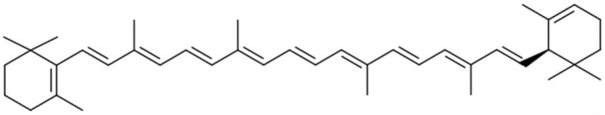
(*3R*)-β-Cryptoxanthin	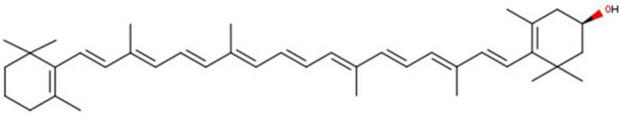
(*3′R*)-β-Cryptoxanthin	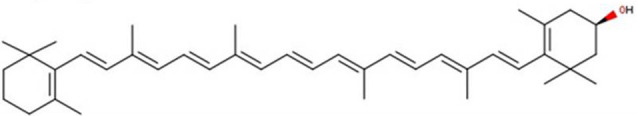
(*3R,3′R*)-Zeaxanthin	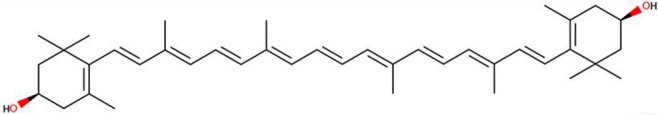
(*3R,3′S, meso*)-Zeaxanthin	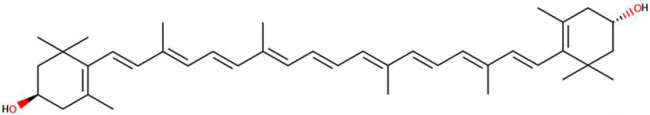
(*3R,3′R*)-Lutein	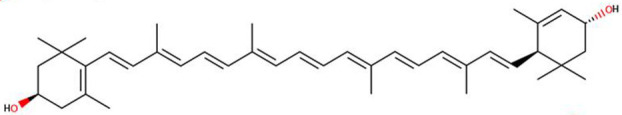
(*3S,3′S*)-Astaxanthin	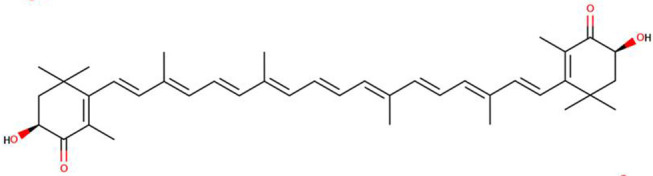
Canthaxanthin	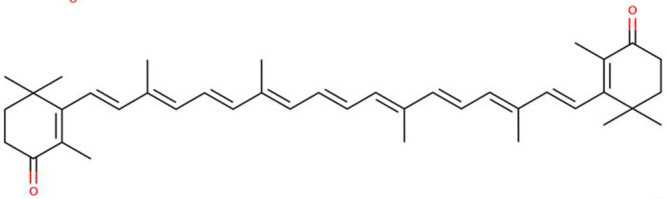
(*3S,3′S*)-Violaxanthin	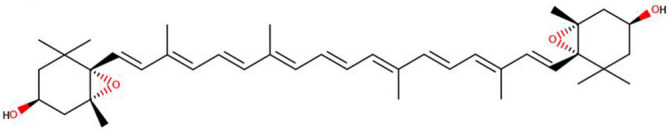
(*all-E*)-Lycopene	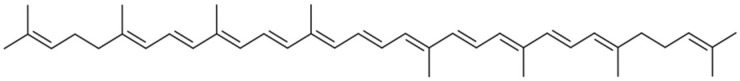
(*5Z*)-Lycopene	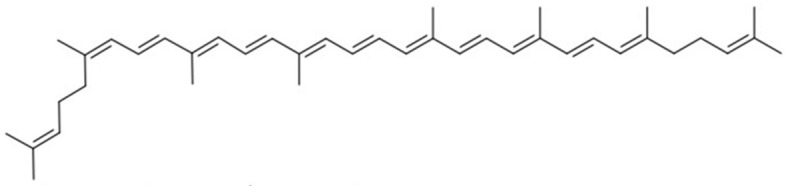
(*5′Z*)-Lycopene	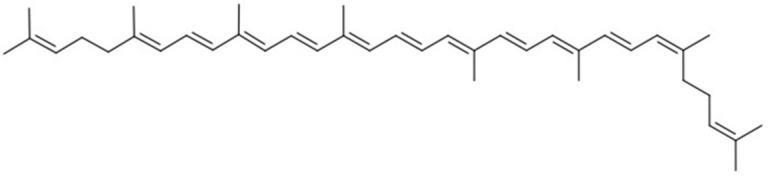
(*15Z*)-Phytofluene	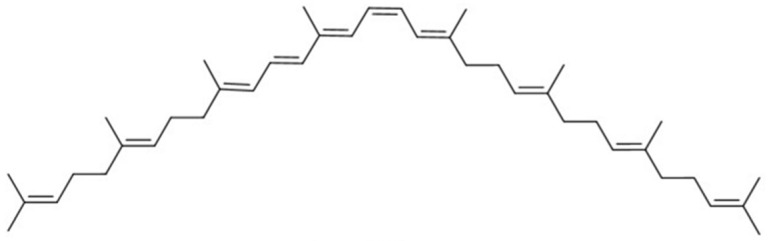
(*15Z*)-Phytoene	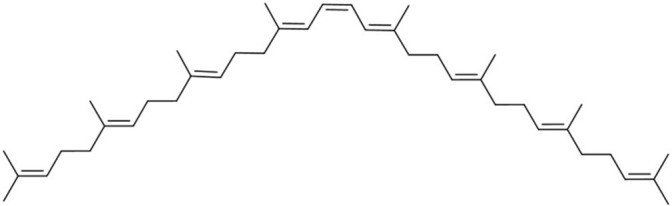

**Table 2:** Average bond distances (Å) with a corresponding standard deviation of the 14, 15, 15′ and 14′ atoms of β-carotene to Fe^2+^ and water molecules TIP2035 and TIP2089, for BCO1.

	C14	C15	C15′	C14′
Fe^2+^	6.65(0.69)	6.35 (0.75)	6.61 (0.66)	6.78 (0.65)
TIP2035	4.87 (0.65)	4.64 (0.72)	4.79 (0.65)	5.18 (0.66)
TIP2089	7.21 (0.69)	6.02 (0.78)	6.53 (0.71)	6.02 (0.72)

**Table 3:** Average bond distances in Å with the corresponding standard deviation of the carbons 8, 9, 10 and 11 of β-carotene to Fe^2+^, and to water molecules TIP1 and TIP1453, for BCO2.

	C8	C9	C10	C11
Fe^2+^	6.65(0.69)	6.35 (0.75)	6.61 (0.66)	6.78 (0.65)
TIP1	4.87 (0.65)	4.64 (0.72)	4.79 (0.65)	5.18 (0.66)
TIP1453	7.21 (0.69)	6.02 (0.78)	6.53 (0.71)	6.02 (0.72)

**Table 4:** System–ligand total potential interaction energy (Uab) and standard deviation (SD), between BCO1 and all carotenoids that were analyzed, for 1 ns of production, in Kcal.mol^−1^. The second column details the average total potential interaction energy. The third and fourth columns show the electrostatic and Van der Waals contributions to Uab, respectively. The fifth column corresponds to the difference in conformational energies. This is the average value of the difference in energies between the minimum and the pose adopted at the binding site at each sampling step.

Carotenoids	Uab (SD)	Elec. (SD)	VDW (SD)	Con. Dif. Ener. (SD)
α-Carotene	−92.20 (3.13)	−5.99 (1.45)	−86.22 (2.98)	63.17 (9.44)
(15*Z*)-Phytoene	−92.97 (3.40)	−5.05 (1.47)	−87.92 (3.09)	68.41 (9.44)
β-Carotene	−94.78 (8,34)	−6.04 (2,19)	−88.76 (3,09)	62.95 (8.73)
(5′*Z*)-Lycopene	−98.54 (3.64)	−5.35 (1.97)	−93.19 (3.01)	67.06 (7.69)
(all-*E*)-Lycopene	−99.91 (3.72)	−7.15 (2.25)	−92.76 (3.17)	67.02 (7.72)
(3′*R*)-β-Cryptoxanthin	−99.95 (3.08)	−10.78 (2.35)	−89.18 (2.57)	64.44 (8.32)
(5*Z*)-Lycopene	−101.13 (4.32)	−7.99 (2.16)	−93.14 (3.65)	65.02 (10.12)
(15*Z*)-Phytofluene	−101.88 (3.98)	−7.55 (2.11)	−94.33 (3.39)	67.25 (9.90)
(3*R*)-β-Cryptoxanthin	−103.99 (4.21)	−13.29 (3.63)	−90.60 (3.35)	63.94 (8.77)
(3*S*,3′*S*)-Violaxanthin	−104.04 (4.07)	−13.96 (3.67)	−90.08 (3.77)	68.12 (9.08)
(3*R*,3′*R*)-Lutein	−107.42 (4.76)	−20.49 (3.89)	−86.93 (3.30)	64.57 (9.53)
(3*R*,3′*S*, *meso*)-Zeaxanthin	−107.61 (4.45)	−18.62 (3.75)	−88.98 (3.07)	65.91 (9.80)
Canthaxanthin	−108.76 (4.86)	−19.61 (3.59)	−89.15 (3.41)	63.13 (9.57)
(3*R*,3′*R*)-Zeaxanthin	−116.24 (7.06)	−26.77 (7.63)	–89.47 (3.36)	67.56 (10.41)
(3*S*,3′*S*)-Astaxanthin	−124.02 (6.79)	−36.01 (7.22)	−88.01 (3.49)	64.14 (8.14)

**Table 5:** Fingerprint analysis for BCO1. The first row lists the individual residues (one-letter coding). The second row describes the nature of the residues: A = non-polar; AAr = non-polar aromatic; P = polar; PAr = polar aromatic; neg = negatively charged; pos = positively charged. ALC = α-carotene; BCR = β-cryptoxanthin; ZEA = (3*R*,3′*R*)-zeaxanthin; MZE = (3*R*,3′*S*, *meso*)-zeaxanthin; LUT = (3*R*,3′*R*)-lutein; CAN = canthaxanthin; AST = (3*S*,3′*S*)-astaxanthin; VXA = (3*S*,3′*S*)-violaxanthin; LIC = lycopene; FTU = (15*Z*)-phytofluene; FIT = (15*Z*)-phytoene. For all carotenoids, the protein residues with which interaction is found at least once during production are marked with a cross. Each cross represents the interaction of each atom interacting with the amino acid residue. In the cases of interaction with the solvent, the number of water molecules with which the ligand interacts is reported (coordination waters with Fe^2+^ are not considered). The numbers in the third column express the percentage of interaction between the residue (columns) and the carotenoid (rows); in all cases, two crosses should also be marked (omitted) except for (3*R*)-BCR and AST, for which only one atom of the ligand interacts with the highest percentage residue.

	H2O	F51	F93	F120	F307	F335	F253	F276	F422	P455	P432	A423	A456	L513	L406	M260	W270	Y332	Y235	Y326	T141	T424	T433	C273	E457	D512	H237	K434	R408
		A Ar	A Ar	A Ar	A Ar	A Ar	A Ar	A Ar	A Ar	A	A	A	A	A	A	A	P Ar	P Ar	P Ar	P Ar	P	P	P	P	P	neg	neg	pos	pos
ALC		-	65	-	-												--										-		
(3R)-BCR	21	-	-	18	-	-										-	-							--					
(3′R)-BCR		-	--	-	-	-				-			75				-												--
ZEA	3	-	--	--		-							--	--			--	-			96	-	-		--	--	-		--
MZE	15	-	-	-									85			--	-						-						
LUT	15	-	--	--	-					99						-	--						--				-		
AST	30	-	-		-					72	-		--			-	-					--	---						
CAN	23	-	-		-											-	-						20				-		
VXA	1	-	--	--	-	-			-		-	-			-		--					--	----				-	-	58
(all-E)-LIC		--	66	-	-	-											-	-											
(5Z)-LIC		--	--	--		-											57	-											
(5′Z)-LIC		-	51	-	-	-											--	-	-										
FTU		34			--	-	-	-										-	-	-							--		
FIT		40			--	-	--																				-		-

**Table 6:** Averages of the system–ligand interaction energies (Uab) in Kcal.mol^−1^ and standard deviation (SD) between BCO2 and the ligands listed in the first column, for 1 ns of production. Total potential interaction energy, electrostatic and Van der Waals contributions are shown. The fifth column corresponds to the difference in conformational energies. This is the average value of the difference in energies between the minimum and the pose adopted at the binding site at each sampling step.

Carotenoids	Uab (SD)	Elec. (SD)	VDW (SD)	Con. Dif. Ener. (SD)
α-Carotene	−86.56 (3.89)	−3.66 (1.79)	−82.92 (3.30)	64.23 (8.15)
(15*Z*)-Phytofluene	−87.82 (4.07)	−4.83 (1.74)	−82.99 (3.43)	63.62 (10.17)
(15*Z*)-Phytoene	−90.58 (4.68)	−5.82 (1.98)	−85.31 (4.16)	68.00 (8.09)
β-Carotene	−91.74 (3.99)	−5.82 (2.22)	−85.92 (3.49)	64.11 (8.06)
(5′*Z*)-Lycopene	−96.67 (3.95)	−6.52 (2.17)	−90.15 (3.48)	64.67 (8.6)
(all-*E*)-Lycopene	−104.16 (4.13)	−10.10 (2.93)	−94.07 (3.14)	66.13 (7.60)
(3′*R*)-β-Cryptoxanthin	−104.16 (4.98)	−19.83 (4.57)	−84.33 (3.29)	63.64 (7.73)
(5*Z*)-Lycopene	−104.39 (3.66)	−7.47 (1.94)	−96.92 (3.18)	64.61 (9.94)
Canthaxanthin	−105.67 (5.88)	−22.70 (5.56)	−82.97 (3.47)	64.35 (9.39)
(3*R*)-β-Cryptoxanthin	−108.92 (4.99)	−18.50 (4.03)	−90.42 (3.53)	64.75 (7.35)
(3*R*,3′*S*, *meso*)-Zeaxanthin	−113.11 (5.53)	−27.30 (5.30)	−85.81 (6.63)	65.65 (10.53)
(3*R*,3′*R*)-Zeaxanthin	−114.79 (5.13)	−29.49 (5.63)	−85.30 (3.89)	66.19 (9.71)
(3*R*,3′*R*)-Lutein	−116.76 (5.46)	−29.56 (5.20)	−87.20 (3.62)	66.54 (8.80)
(3*S*,3′*S*)-Violaxanthin	−118.04 (5.61)	−33.96 (6.10)	−84.08 (4.04)	66.84 (12.42)
(3*S*,3′*S*)-Astaxanthin	−126.90 (8.09)	−38.21 (8.48)	−88.70 (4.31)	66.46 (7.13)

**Table 7:** PLIF results for BCO2. The first row lists the residues (one-letter coding). The second row describes the nature of the residues: A = non-polar; AAr = non-polar aromatic; P = polar; PAr = polar aromatic; neg = negatively charged; pos = positively charged. ALC = α-carotene; BCR = β-cryptoxanthin; ZEA = (3*R*,3′*R*)-zeaxanthin; MZE = (3*R*,3′*S*, *meso*)-zeaxanthin; UT = (3*R*,3′*R*)-lutein; CAN = canthaxanthin; AST = (3*S*,3′*S*)-astaxanthin; VXA = (3*S*,3′*S*)-violaxanthin; LIC = lycopene; FTU = (15*Z*)-phytofluene; FIT = (15*Z*)-phytoene. For all carotenoids, the various protein residues with which interaction is found for at least one time point during production are marked with a cross. Each cross represents the interaction of each atom interacting with the amino acid residue. In the cases of interaction with solvent, it is reported how many water molecules the ligand interacts with. The numbers in the third column express the percentage of interaction between the residue (columns) and the carotenoid (rows). In all cases, two crosses should also be marked (omitted) except for (3*R*,3′*R*)-lutein and (3*S*,3′*S*)-astaxanthin where the highest frequency is with water (48% and 36%, respectively).

	H_2_O	F103	F145	F242	F319	F356	F465	F486	F572	P285	G489	G169	L489	W102	Y384	Y284	N221	N384	R315	K174	Q375	H488	H286	H357	H537	H400	E382	E464
		A Ar	A Ar	A Ar	A Ar	A Ar	A Ar	A Ar	A Ar	A	A	A	A	P Ar	P Ar	P Ar	P	P	pos	pos	neg	neg	neg	neg	neg	neg		
ALC		--	-	-	45	--	-	-	--					-		-							--	-				
(3R)-BCR	41	-	-		57	-		-		-								-	--									
(3′R)-BCR	16	-	-		47		---	--		-													-	-			-	
ZEA	39	-	-		-	-		-		-		--							24			--	-			-		
MZE	37		-		-	-		-		-			--			-			46				-			--		--
LUT	45	--	-		-	-	-	-											--				--			--		
AST	24	-			--	-	-	-	-				-			-											-	
CAN	47	--	-		-	-		-		-							-		--		--		-				30	
VXA	62	33	-		-	-	-	--		-	-			--		-	-			-			--				--	
(all-E)-LIC		--	--	-	69	--		-	-	-						-							-	-				
(5Z)-LIC		36	--													-												
(5′Z)-LIC		--	-	-	--	--	25	-	-	-					--	-							-		-			
FTU		-	-		-	50	-	-							-	-												
FIT		-	-		45	--	-	-		-						--							-	-				

**Table 8:** Stages of 100 ns molecular dynamics simulation. Prior to production, the system was prepared, with four stages in which restraints were maintained. nStep = sampling points corresponding to 2 fs. Freq = every how many fs values are noted.

Stages	nStep	Freq	Restraints	Ensamble	T (°C)	P (atm)
First minimization	200,000	1000	backbone, ligand, Fe^2+^, coordination sphere, residues at 10 Å of ligand	-	-	1
Second minimization	20,000	1000	backbone	-	-	1
Annealing	144,000	1000	backbone	NPT	27	1
Equilibration	500,000	1000	backbone	NPT	27	1
Production	50,000,000	100,000	no	NPT	27	1

## Data Availability

All data are available by request.

## Supplementary Materials

The following supporting information can be downloaded at: https://www.mdpi.com/article/10.3390/molecules27227813/s1, Figure S1: Primary structure of BCO1; Figure S2: Primary structure of BCO2; Table S1: Sequences producing significant alignment with BCO1 according to the BLAST platform; Table S2: Sequences producing significant alignment with BCO1 according to the SAS platform; Table S3: Results of homology modeling of BCO1; Table S4: Results of homology modeling of BCO2; Figure S3: Schematic overlay of the amino acid residue sequences of the gen and the three-dimensional model of BCO1; Figure S4: Schematic overlay of the amino acid residue sequences of the gen and the three-dimensional model of BCO2; Figure S5: Superposition of secondary structures for BCO1 and BCO2 after homology modeling; Table S5: Percentage of residues conforming each type of secondary structure over the total number of residues for both enzymes, after homology modeling; Figure S6: Fluctuations of the binding distances of carbons 15, 15′, 14 and 14′ of β-carotene to Fe^2+^ and to the water molecule TIP2035, during the whole simulation, for BCO1; Figure S7: Fluctuations of the binding distances of carbons 8, 9, 10 y 11 of β-carotene to Fe^2+^ and to the water molecule TIP2035, during the whole simulation, for BCO2; Figure S8: RMSD along the entire production path for the BCO1 system; Figure S9: Total energy of the BCO1 system with β-carotene and solvent, during the 100 ns of simulation; Figure S10: Results of RMSF analysis for BCO1; Figure S11: Plots of the components contributing to the total energy for the entire BCO1 simulation; Figure S12: Results of RMSF analysis for BCO2; Figure S13: RMSD along the entire production path for the BCO2 system; Figure S14: Total energy of the BCO2 system with β-carotene and solvent, during the 100 ns of simulation; Figure S15: Plots of the components contributing to the total energy for the entire BCO2 simulation; Figure S16: Plots of the potential interaction energy of BCO1 with β-carotene (Uab); Figure S17: Statistical analysis of the interaction frequency of BCO1 with β-carotene, according to PLIF analysis; Figure S18: Plots of the potential interaction energy of BCO2 with β-carotene; Figure S19: Statistical analysis of the interaction frequency of BCO2 with β-carotene, according to PLIF analysis.

## Sample Availability

Not available.
