# Supplementary material for: A Computer Simulation Insight into the Formation of Apocarotenoids: Study of the Carotenoid Oxygenases BCO1 and BCO2 and Their Interaction with Putative Substrates
Source: Molecules. 2022 Nov 13;27(22):7813. doi: 10.3390/molecules27227813 (PMC9693266; doi:10.3390/molecules27227813)
Supplement: Supplementary file 1 [file molecules-27-07813-s001.zip › molecules-1916893-supplementary.pdf]

SUPPLEMENTARY MATERIAL

A Computer Simulation Insight into the Formation of Apocarotenoids: Study of the Carotenoid Oxygenases BCO1 and BCO2 and Their Interaction with Putative Substrates

Anabela Martínez [1,†], Jorge Cantero [1,2,†], Antonio J. Meléndez-Martínez [3,*] and Margot Paulino [2,*]

[1] Bioinformatics Area, DETEMA Department, Faculty of Chemistry, UdelaR, Montevideo 11600, Uruguay
[2] Medical Research Center, Faculty of Health Sciences, Universidad Nacional del Este, Minga Guazu 7420, Paraguay
[3] Food Color and Quality Laboratory, Faculty of Pharmacy, Universidad de Sevilla, 41012 Sevilla, Spain
* Correspondence: ajmelendez@us.es (A.J.M.-M.); margot@fq.edu.uy (M.P.)
† These authors contributed equally to this work.

```
   10         20         30         40         50
MDIIFGRNRK EQLEPVRAKV TGKIPAWLQG TLLRNGPGMH TVGESRYNHW
       60         70         80         90        100
FDGLALLHSF TIRDGEVYYR SKYLRSDTYN TNIEANRIVV SEFGTMAYPD
      110        120        130        140        150
PCKNIFSKAF SYLSHTIPDF TDNCLINIMK CGEDFYATSE TNYIRKINPQ
      160        170        180        190        200
TLETLEKVDY RKYVAVNLAT SHPHYDEAGN VLNMGTSIVE KGKTKYVIFK
      210        220        230        240        250
IPATVPEGKK QGKSPWKHTE VFCSIPSRSL LSPSYYHSFG VTENYVIFLE
      260        270        280        290        300
QPFRLDILKM ATAYIRRMSW ASCLAFHREE KTYIHIIDQR TRQPVQTKFY
      310        320        330        340        350
TDAMVVFHHV NAYEEDGCIV FDVIAYEDNS LYQLFYLANL NQDFKENSRL
      360        370        380        390        400
TSVPTLRRFA VPLHVDKNAE VGTNLIKVAS TTATALKEED GQVYCQPEFL
      410        420        430        440        450
YEGLELPRVN YAHNGKQYRY VFATGVQWSP IPTKIIKYDI LTKSSLKWRE
      460        470        480        490        500
DDCWPAEPLF VPAPGAKDED DGVILSAIVS TDPQKLPFLL ILDAKSFTEL
      510        520        530        540
ARASVDVDMH MDLHGLFITD MDWDTKKQAA SEEQRDRASD CHGAPLT
```

Figure S1. Primary structure of BCO1.

```
       10         20         30         40         50
MFFRVFLHFI RSHSATAVDF LPVMVHRLPV FKRYMGNTPQ KKAVFGQCRG
       60         70         80         90        100
LPCVAPLLTT VEEAPRGISA RVWGHFPKWL NGSLLRIGPG KFEFGKDKYN
      110        120        130        140        150
HWFDGMALLH QFRMAKGTVT YRSKFLQSDT YKANSAKNRI VISEFGTLAL
      160        170        180        190        200
PDPCKNVFER FMSRFELPGK AAAMTDNTNV NYVRYKGDYY LCTETNFMNK
```

```
      210        220        230        240        250
VDIETLEKTE KVDWSKFIAV NGATAHPHYD LDGTAYNMGN SFGPYGFSYK
      260        270        280        290        300
VIRVPPEKVD LGETIHGVQV ICSIASTEKG KPSYYHSFGM TRNYIIFIEQ
      310        320        330        340        350
PLKMNLWKIA TSKIRGKAFS DGISWEPQCN TRFHVVEKRT GQLLPGRYYS
      360        370        380        390        400
KPFVTFHQIN AFEDQGCVII DLCCQDNGRT LEVYQLQNLR KAGEGLDQVH
      410        420        430        440        450
NSAAKSFPRR FVLPLNVSLN APEGDNLSPL SYTSASAVKQ ADGTIWCSHE
      460        470        480        490        500
NLHQEDLEKE GGIEFPQIYY DRFSGKKYHF FYGCGFRHLV GDSLIKVDVV
      510        520        530        540        550
NKTLKVWRED GFYPSEPVFV PAPGTNEEDG GVILSVVITP NQNESNFILV
      560        570
LDAKNFEELG RAEVPVQMPY GFHGTFIPI
```

Figure S2. Primary structure of BCO2.

Table S1. Sequences producing significant alignment with BCO1 according to the BLAST platform and main parameters of the respective crystals. COD= PDB identification code; DESC=description of protein type; %Id = percent identity; ORG = organism in which expressed; CLASS = classification according to enzymatic function; LIG&Ion = co-crystallized ligands and ions; R2 = resolution expressed in A.

COD	DESC	%Id	ORG	FUNCTION	CLASS	LIG&Ion	R^2
3FSN	RPE65	39.50	Bos Tauro	Retinal pigment epithelium specific protein.	Isomerase	Tetraethylene glycol, Fe^{2+}	2.14
4RSE	RPE65	39.50	Bos Tauro	Retinal pigment epithelium specific protein.	Isomerase	Phenylpropane, Palmitic acid, Fe^{2+}	2.39
6VCF	Nitrosotalea devanaterra dioxygenase	36.38	Escherichia Coli	carotenoid cleavage dioxygenase	Oxide reductase	Bicarbonate ion, Fe^{2+}, Cl^-, Na^+	2.69
6C7O	RPE65 chimera	29.34	Escherichia Coli	Apocarotenoid-15,15'-oxygenase	Oxide reductase	Fe^{2+}	3.15
6C7K	RPE65 chimera	29.15	Escherichia Coli	Apocarotenoid-15,15'-oxygenase	Oxide reductase	Fe^{2+}, Cl^-	2.50
5KJA	ACO mutant	28.96	Synechocystis	Apocarotenoid-15,15'-oxygenase	Oxide reductase	Fe^{2+}, Cl^-	2.80
2BIW	apocarotenoid cleavage oxygenase	28.96	Synechocystis	Apocarotenoid-cleaving'-oxygenase	Oxide reductase	Hydroxyapocarotenol, Fe^{2+}	2.39
5KJD	ACO	28.76	Synechocystis	Apocarotenoid-15,15'-oxygenase	Oxide reductase	Fe^{2+}	2.75
5KJB	ACO mutant	28.76	Synechocystis	Apocarotenoid-15,15'-oxygenase	Oxide reductase	Fe^{2+}	2.81
5KK0	ACO mutant	28.76	Synechocystis	Apocarotenoid-15,15'-oxygenase	Oxide reductase	Fe^{2+}, Cl^-	2.80

Table S2. Sequences producing significant alignment with BCO1 according to the SAS platform. COD= PDB identification code; DESC=description of protein type; %Id = percent identity; ORG = organism in which expressed; CLASS = classification according to enzymatic function; LIG&Ion = ligands and co-crystallized ions; R2 = resolution expressed in A.

COD	DESC	%Id	ORG	FUNCTION	CLASS	LIG&Ion	R^2

4F30	RPE65 cultivated in ammonium phosphate solution	38.0	Bos Taurus	Retinoid isomer hydrolase	Isomerase, Hydrolase	$PO4^{2-}$, Fe^{2+}	3.15
6VCG	Candidatus Nitrosotalea devanaterra	35.8	Candidatus Nitrosotalea devanaterra	carotenoid cleavage dioxygenase	Oxidoreduc tase	Co^{2+}, Cl^{-}, Na^{+}	2.30
4RYX	RPE65	37.8	Bos Taurus	Retinoid isomer hydrolase	Isomerase	Amino Phenyl Propanol, Palmitic acid, Methyl Pentanediol, $SO4^{2-}$, Fe^{2+}	2.00
4F2Z	RPE65 in a lipid environment	37.6	Bos Taurus	Retinoid isomer hydrolase	Isomerase, Hydrolase	Fe^{2+}	3.00
5ULG	RPE65	37.5	Bos Taurus	Retinoid isomer hydrolase	Isomerase, isomerase inhibitor	Aminophenyl Ethanol, Palmitic acid, tetraethylene glycol, Fe^{2+}, Na^{+}	2.10
4RSE	RPE65	37.4	Bos Taurus	Retinoid isomer hydrolase	Isomerase	Aminophenyl Ethanol, Palmitic acid, Fe^{2+}	2.39
4RYY	RPE65	37.4	Bos Taurus	Retinoid isomer hydrolase	Isomerase	Aminophenyl Ethanol, Palmitic acid, Fe^{2+}	2.30
4RYZ	RPE65	37.4	Bos Taurus	Retinoid isomer hydrolase	isomerase	Aminofenoxihexanol, Ácido palmítico, Fe^{2+}	2.50
4ZHK	RPE65	37.4	Bos Taurus	Retinoid isomer hydrolase	Hydrolase, Isomerase	Aminophenyl Ethanol, Tetraethylene glycol, Fe^{2+}, Na^{+}	2.09

Table S3. Results of homology modeling of BCO1 were performed in MOE with the 4ryx crystal as a template. The model selected (the one with the lowest potential energy) was 4.

modelo	RMSD to mean	CA RMSD to mean	Contact Energy	Packing Score	GB/VI	U	E sol	E ele	E vdw	E bond
1	0.53	0.37	-464.32	2.29	-76463.93	-19333.09	-3137.03	-28695.54	890.17	8406.64
2	0.60	0.47	-461.99	2.35	-96433.58	-18109.42	-6124.73	-28717.91	1486.20	8756.27
3	0.56	0.43	-460.43	2.32	-76453.69	-19844.08	-6258.15	-28702.27	572.98	8047.50
4	0.50	0.31	-467.45	2.30	-76493.98	-20775.92	-5799.98	-28941.52	576.72	7442.58
5	0.54	0.37	-456.77	2.33	-76385.23	-19660.26	-6032.83	-28621.32	744.05	8075.31
6	0.55	0.39	-473.70	2.32	-76484.48	-18733.43	-6016.28	-28751.29	9569.12	8985.46
7	0.54	0.37	-466.46	2.32	-76484.61	-15936.77	-5967.64	-28741.01	2117.80	10627.05
8	0.53	0.35	-460.83	2.23	-76597.70	-20557.29	-5602.41	-29101.64	446.66	7845.45
9	0.57	0.44	-453.90	2.27	-76456.99	-20269.98	-6123.93	-28773.63	624.39	7794.89
10	0.48	0.33	-464.23	2.28	-76419.09	–14409.69	-5820.46	-28844.98	2718.83	11639.07

Table S4. Results of homology modeling of BCO2 performed in MOE with the 4ryx crystal as a template. The model selected (the one with the lowest potential energy) was 1.

modelo	RMSD to mean	CA RMSD to mean	Contact Energy	Packing Score	GB/VI	U	E sol	E ele	E vdw	E bond
1	0.66	0.56	-420.81	2.22	-23313.11	-6962.95	-2828.25	-12359.35	-2398.78	7795.18
2	0.71	0.63	-413.55	2.28	-22680.12	-4136.17	-4741.14	-9937.63	-2177.66	7979.11
3	0.69	0.63	-420.90	2.29	-22667.98	-4005.14	-4734.37	-9942.83	-2034.34	7972.03
4	0.64	0.56	-428.21	2.33	-22662.46	-3952.55	-4534.84	-9944.39	-2153.48	8145.32
5	0.60	0.50	-421.04	2.29	-22640.50	-3952.39	-5196.08	-9775.77	-2204.31	8028.18

6	0.62	0.52	-424.77	2.31	-22593.07	-3458.40	-5135.70	-9657.41	-2106.33	8305.34
7	0.65	0.57	-422.89	2.33	-22580.55	-3688.22	-5052.11	-9669.02	-2172.07	8152.87
8	0.69	0.61	-429.13	2.32	-22539.98	-3096.37	-5306.93	-9510.36	-1923.06	8337.05
9	0.67	0.59	-421.54	2.33	-22519.15	-3164.98	-5479.46	-9267.71	-1959.91	8062.65
10	0.65	0.56	-419.27	2.34	-22468.86	-2792.45	-5586.58	-9225.17	-1865.84	8298.50

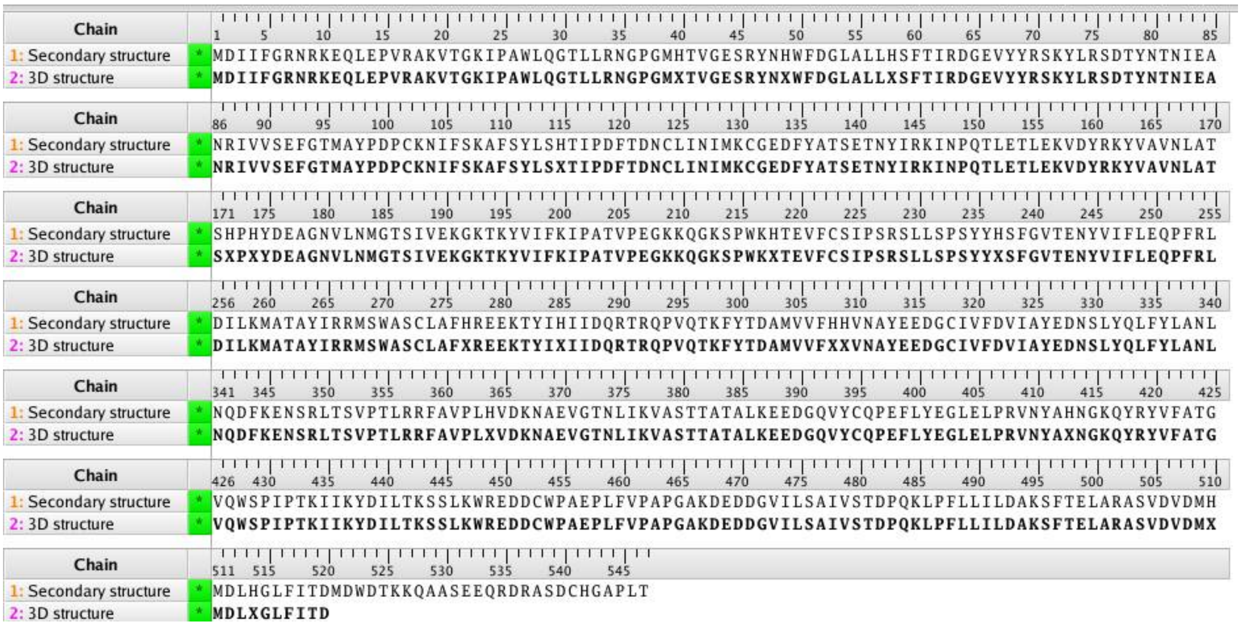

Figure S3. Schematic overlay of the amino acid sequences of the gen (secondary structure) and the three-dimensional model generated by homology modeling for BCO1. It can be seen that the unmodeled regions correspond to the N-terminal region.

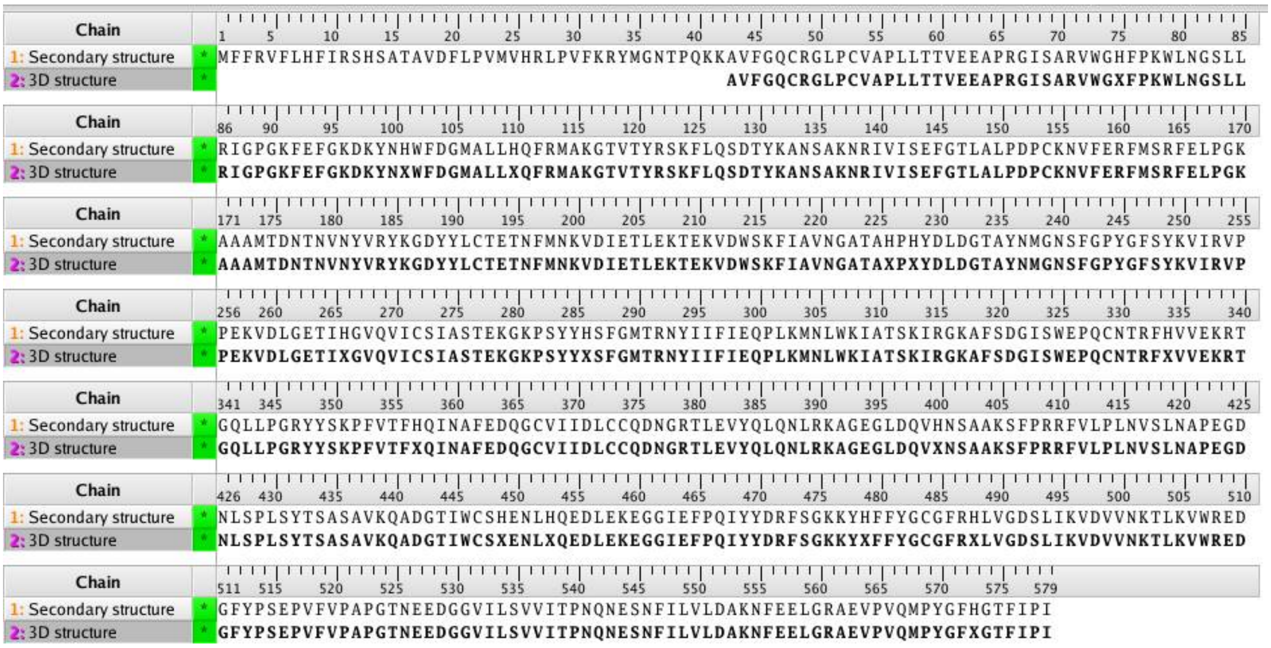

Figure S4. Schematic overlay of the amino acid sequences of the gen and the three-dimensional model generated by homology modeling for BCO2. It can be seen that the unmodeled regions correspond to the C-terminal region.

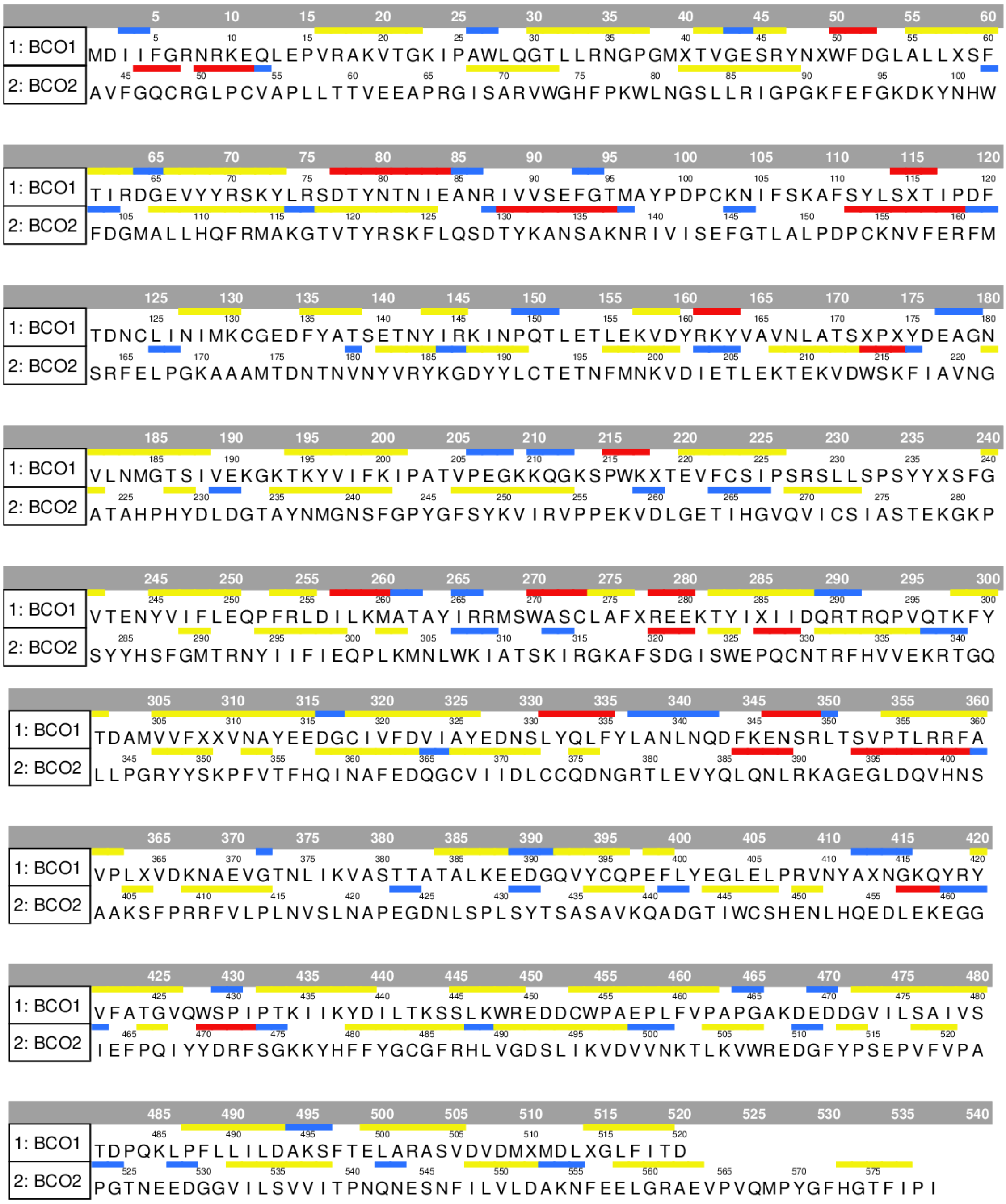

Figure S5. Superposition of secondary structures for BCO1 and BCO2 after homology modeling. On the residues the secondary structure is colored according to: in white random coil, in yellow extended strand, in red alpha helix, in blue beta-turn.

Table S5. Percentage of residues conforming each type of secondary structure over the total number of residues for both enzymes, after homology modeling.

	BCO1	BCO2
random coil	43.27	42.09
extended strand	38.27	35.94
alpha hélix	7.69	9.49
beta turn	10.77	12.48

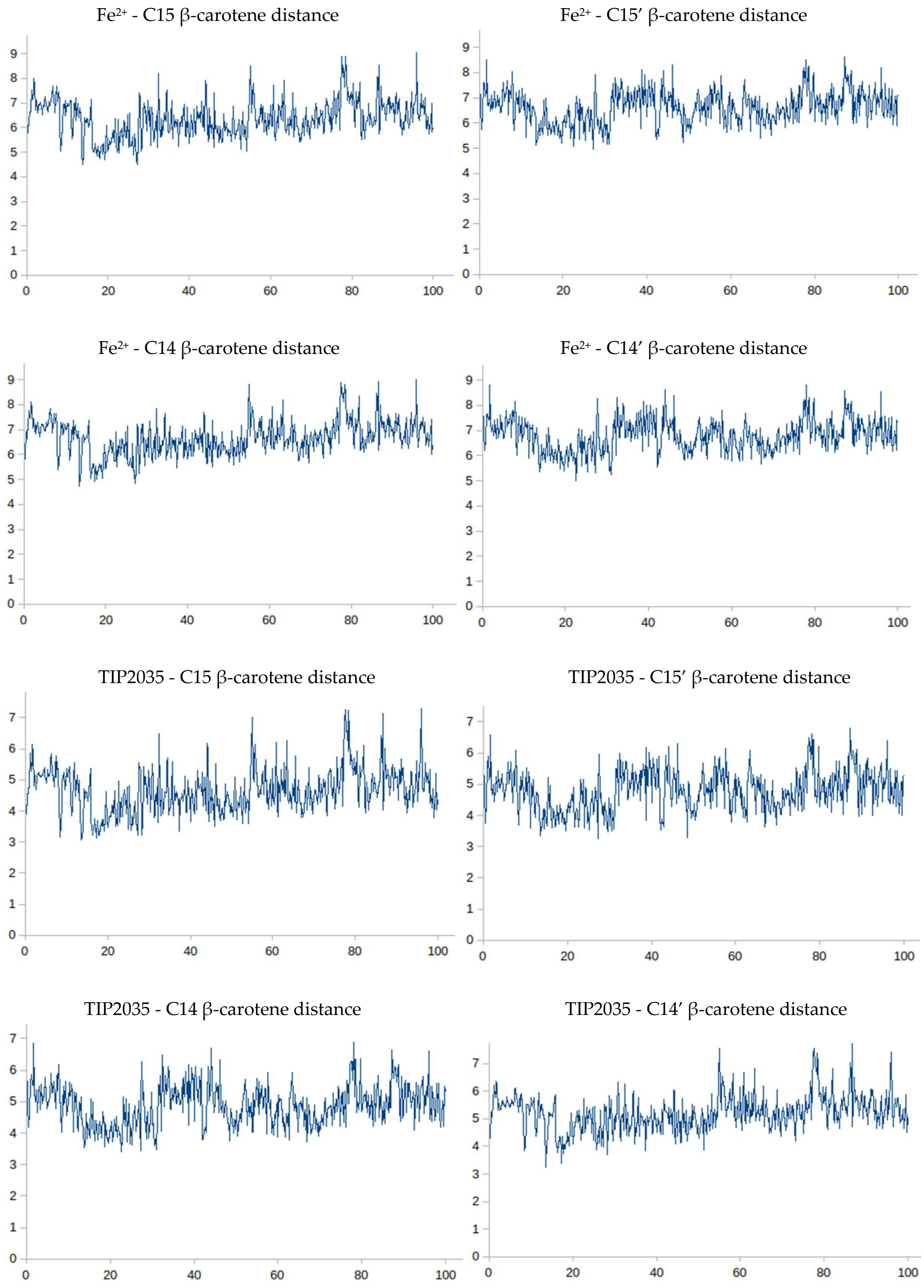

Figure S6. Fluctuations of the binding distances of carbons 15, 15', 14 and 14' of β-carotene to Fe^{2+} and to the water molecule TIP2035, during the whole simulation, for BCO1. All graphs are distance (Å) vs. time (nano seconds).

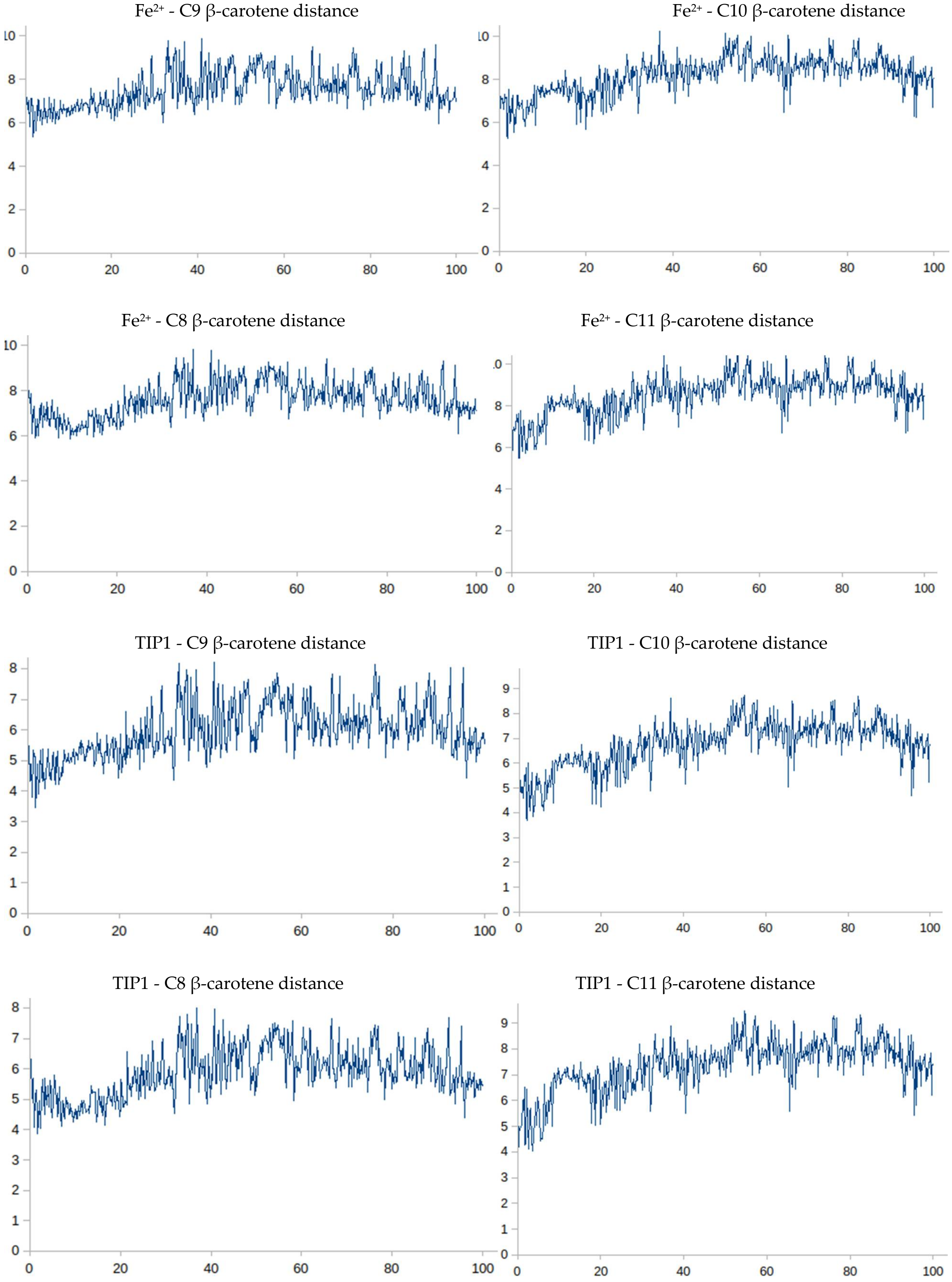

Figure S7. Fluctuations of the binding distances of carbons 8, 9, 10 y 11 of β-carotene to Fe^{2+} and to the water molecule TIP2035, during the whole simulation, for BCO2. All graphs are distance (Å) vs. time (nano seconds).

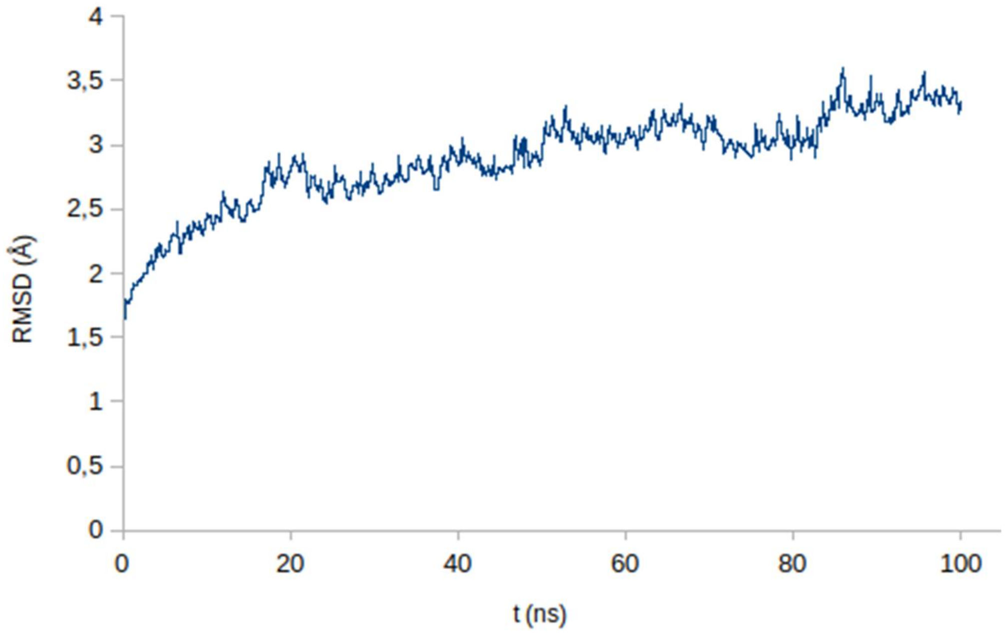

Figure S8. RMSD in Å along the entire production path for the BCO1 system.

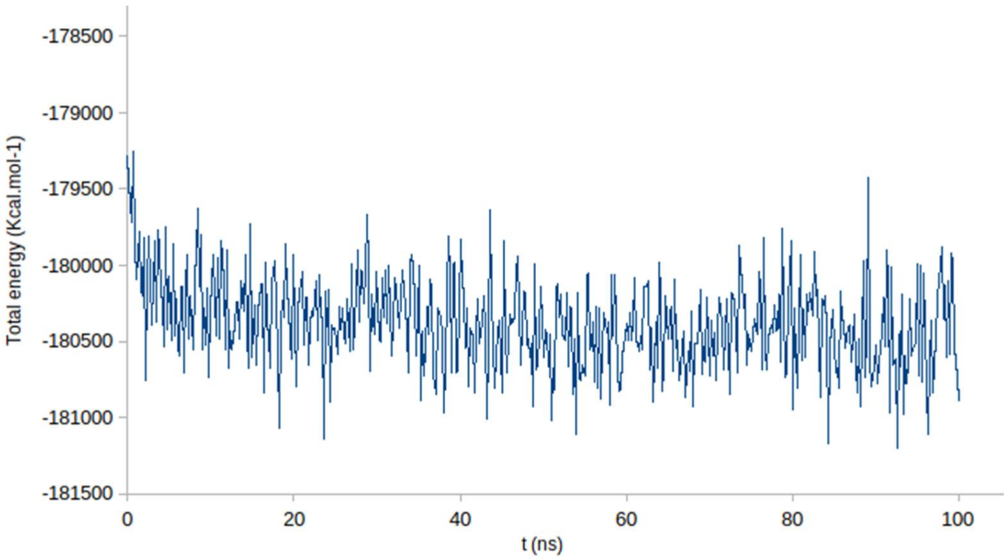

Figure S9. Total energy of the BCO1 system with β-Carotene and solvent, in Kcal.mol^{-1}, during the 100 ns of simulation.

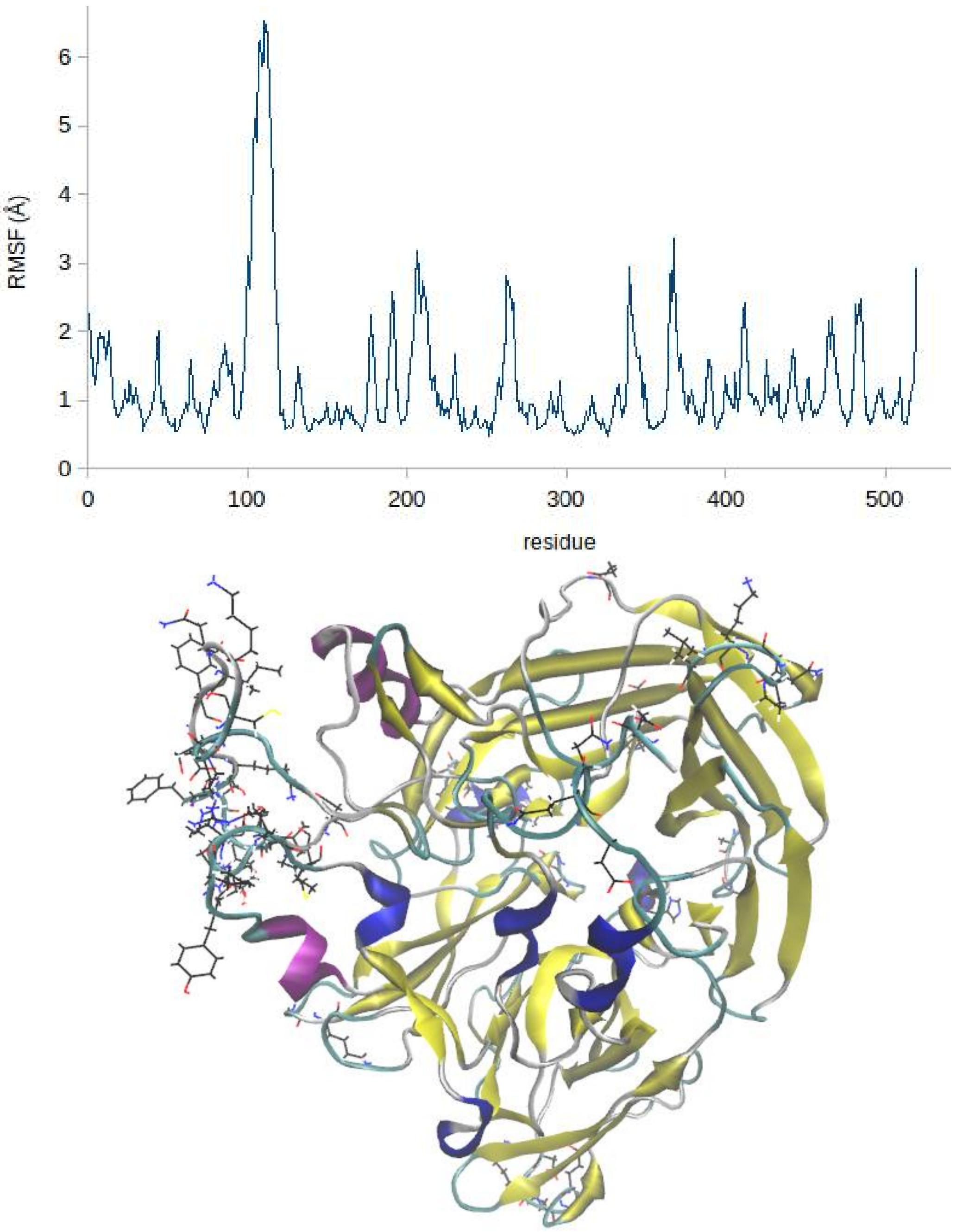

Figure S10. Results of RMSF analysis for BCO1. The image above shows the RMSF plot in Å for all the residues of the protein. The image below shows a two-dimensional schematic of the protein where the residues with RMSF > 2 Å are marked with a drawing of rods, colored according to atom type.

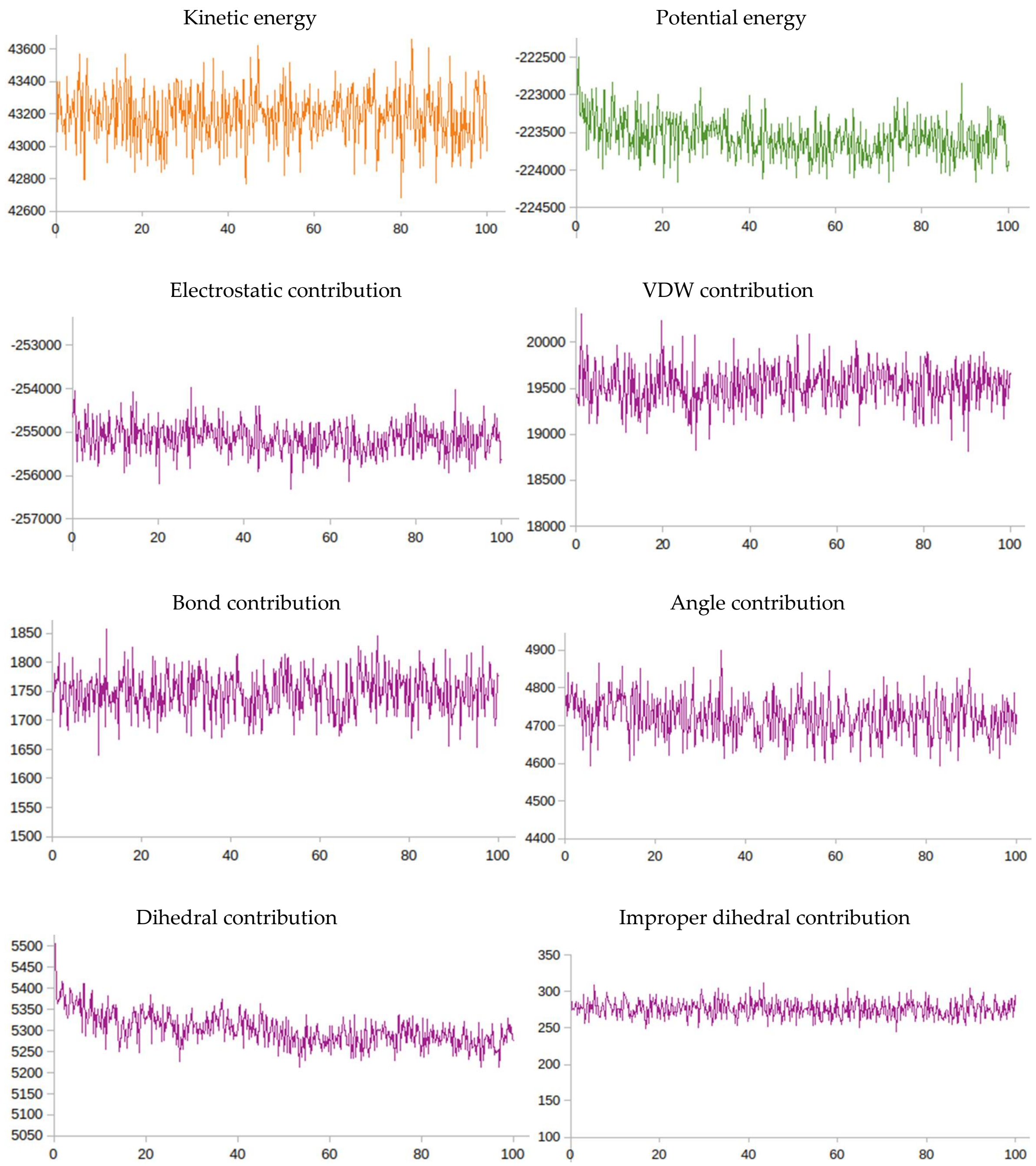

Figure S11. Plots of the components contributing to the total energy for the entire BCO1 simulation. All graphs are Energy (Kcal.mol^{-1}) vs. time (nano seconds).

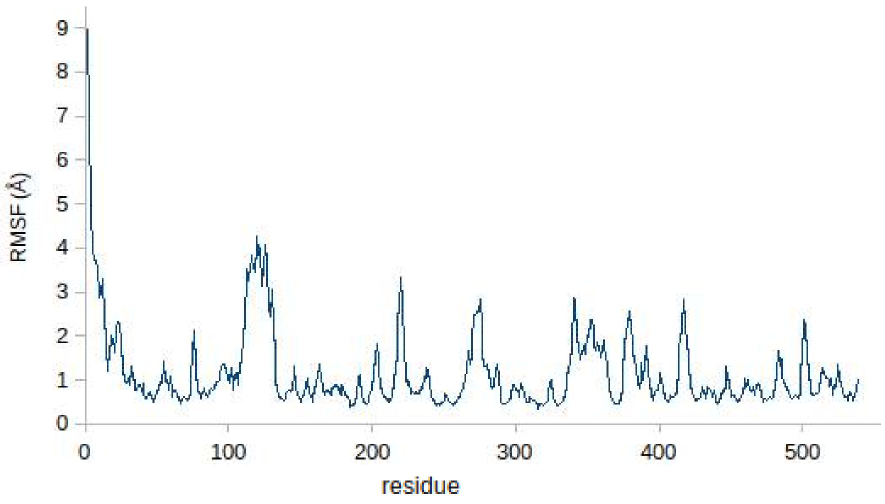

Figure S12. RMSF in Å for all the residues of the protein for BCO2.

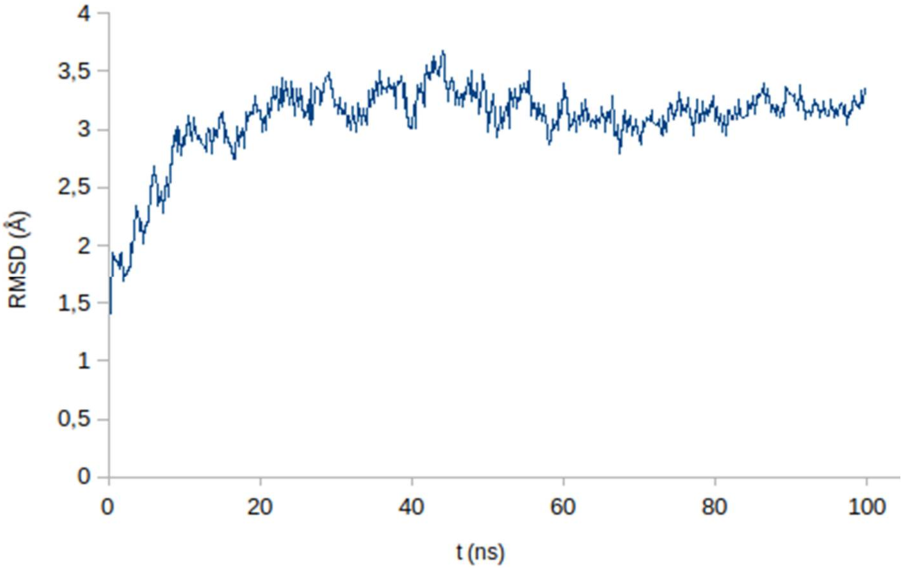

Figure S13. RMSD in Å along the entire production path for the BCO2 system.

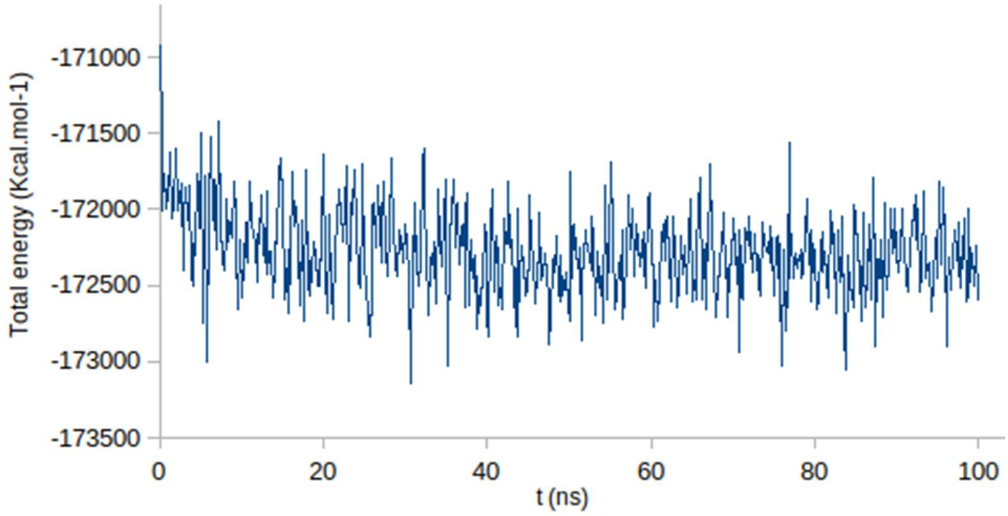

Figure S14. Total energy of the BCO2 system with β-Carotene and solvent, in Kcal.mol^{-1}, during the 100 ns of simulation.

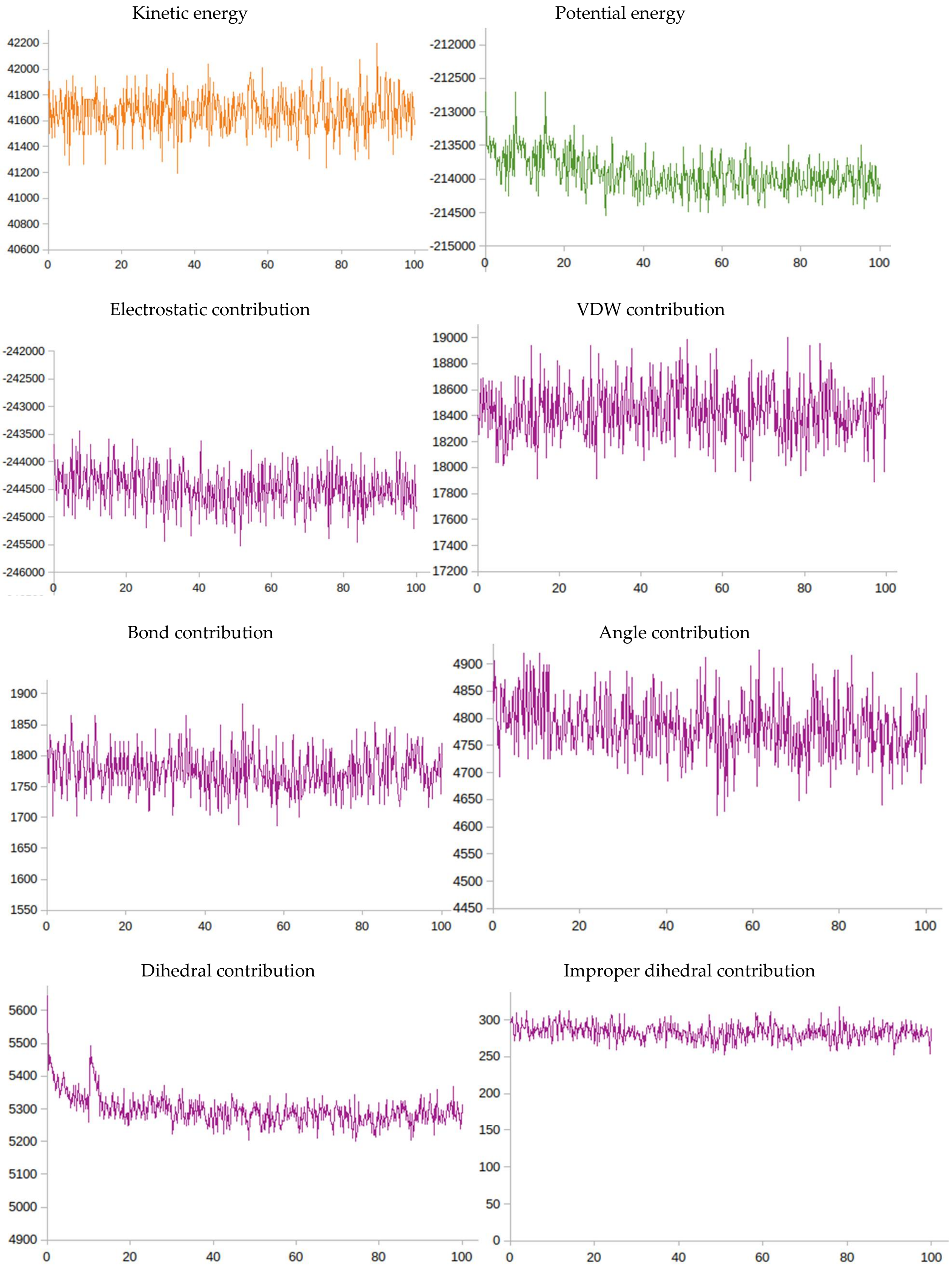

Figure S15. Plots of the components contributing to the total energy for the entire BCO2 simulation. All graphs are Energy (Kcal.mol^{-1}) vs. time (nano seconds).

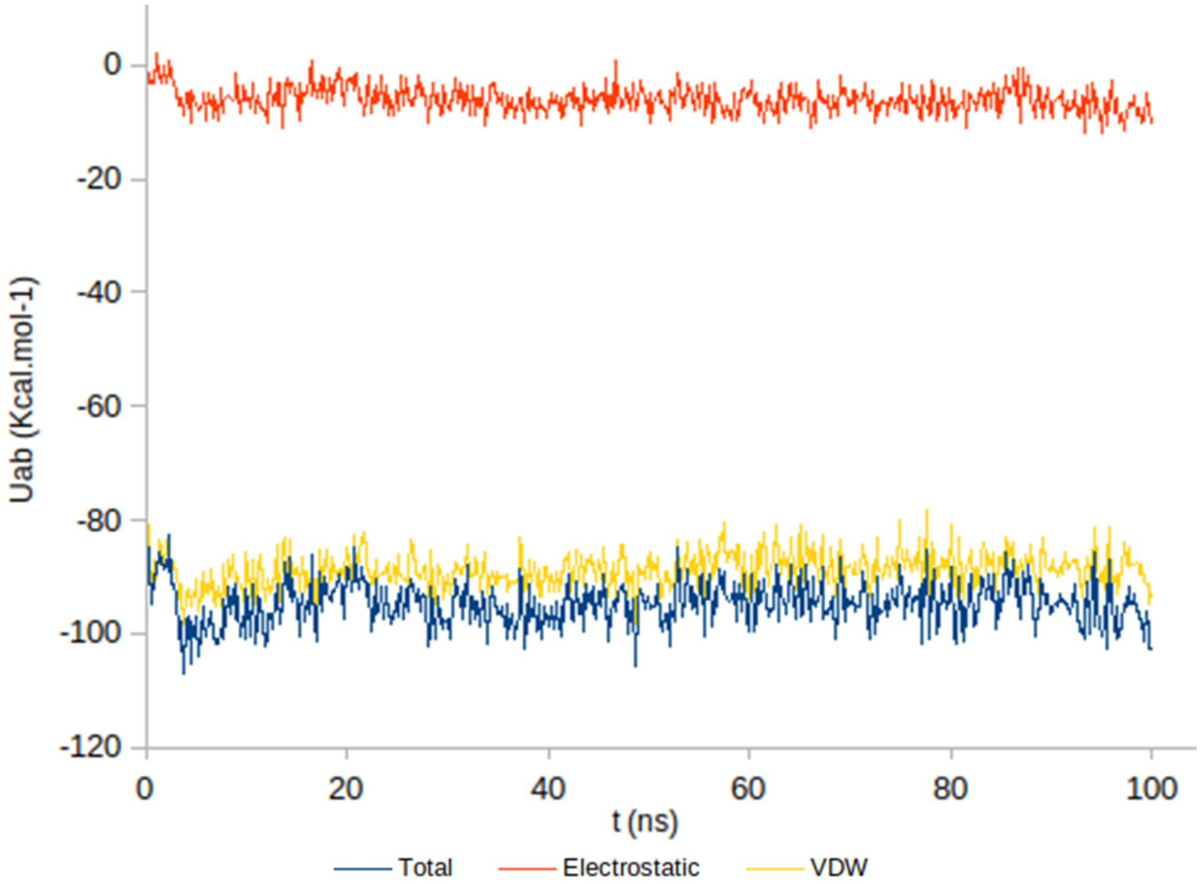

Figure S16. Plots of the interaction energy of BCO1 with β-Carotene (Uab), in Kcal.mol^{-1}. The total energy is plotted in blue. In orange and yellow are the electrostatic and Van der Waals contributions (VDW), respectively.

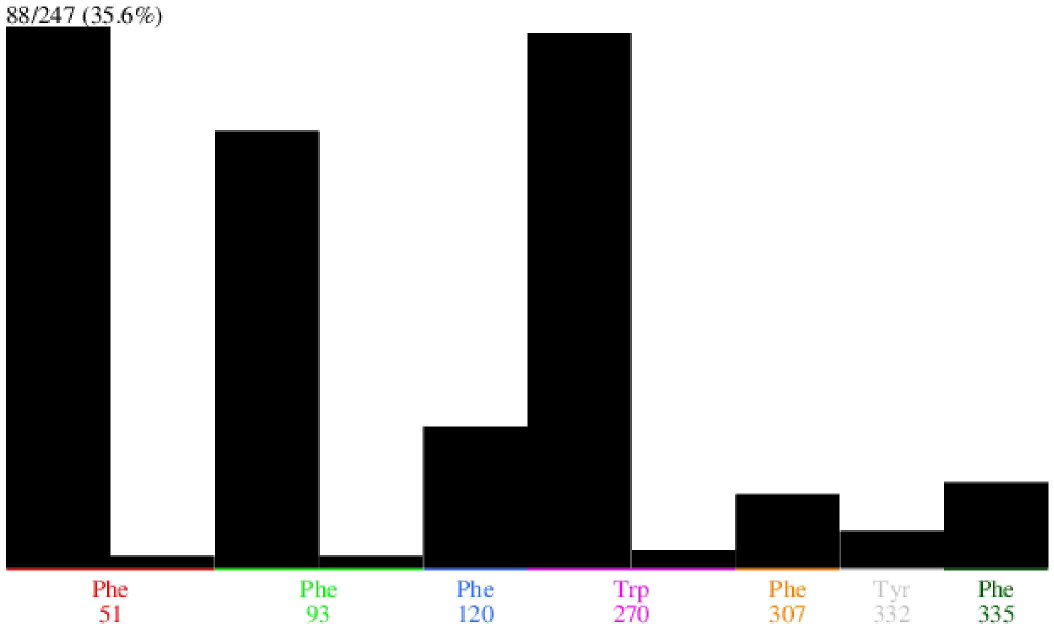

Figure S17. Statistical analysis of the interaction frequency of BCO1 with β-carotene, according to PLIF analysis.

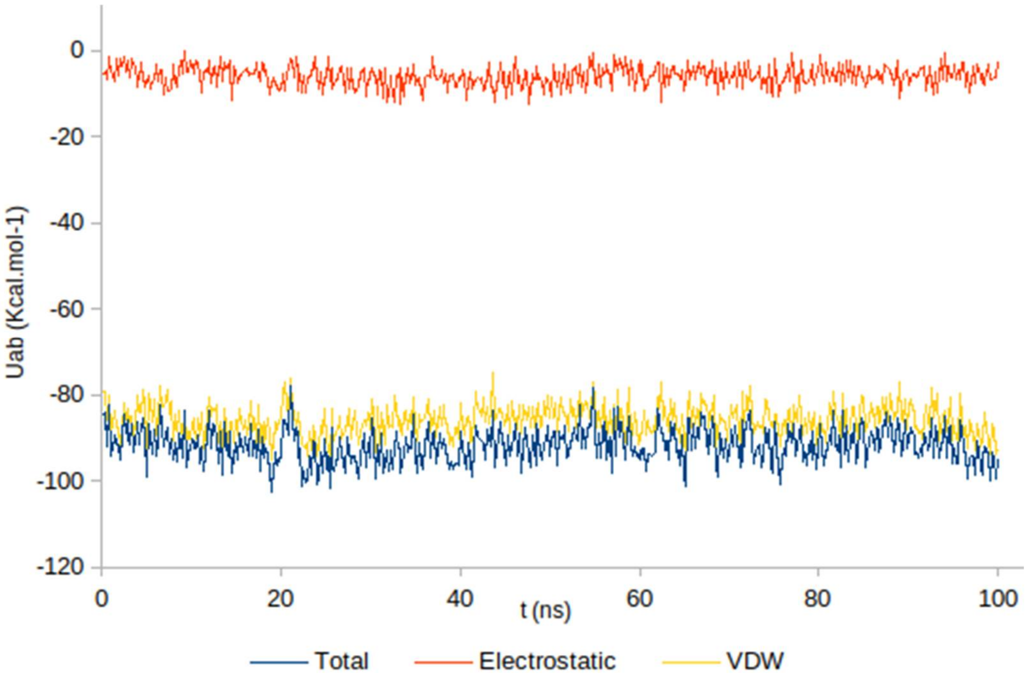

Figure S18. Plots of the interaction energy of BCO2 with β-Carotene (Uab), in Kcal.mol^{-1}. The total energy is plotted in blue. In orange and yellow are the electrostatic and Van der Waals contributions (VDW), respectively.

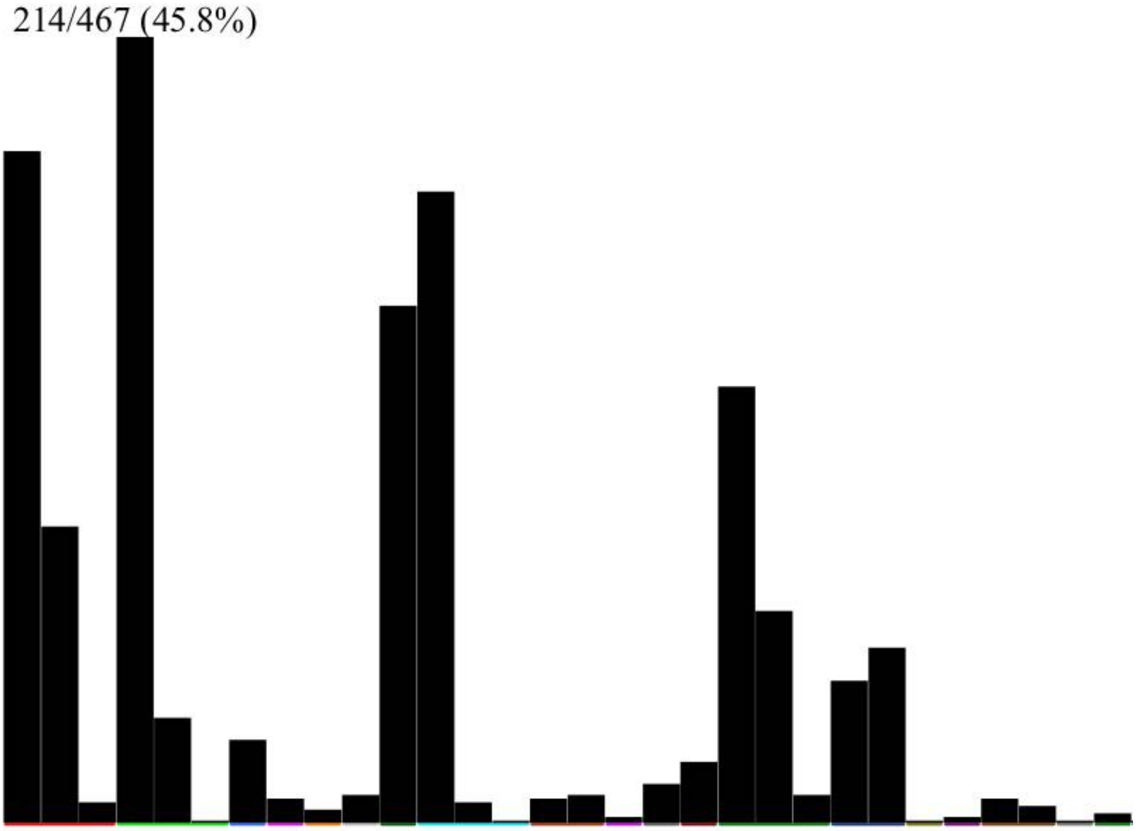

Figure S19. Statistical analysis of the interaction frequency of BCO2 with β-carotene, according to PLIF analysis.
